# Lectin pathway of complement in SLE: MAP-1 as a marker of haematological manifestations and elevated type I interferon activity

**DOI:** 10.1136/lupus-2025-001890

**Published:** 2026-04-09

**Authors:** Linnea Lindelöf, Peter Garred, Mun-Gwan Hong, Sasha Wahl Vælum, Lotte Holten Petersen, Dag Leonard, Ahmed Sayadi, Vilija Oke, Timothy B Niewold, Lina-Marcela Diaz-Gallo, Saedis Saevarsdottir, Iva Gunnarsson, Elisabet Svenungsson, Oskar Eriksson

**Affiliations:** 1 Department of Immunology, Genetics and Pathology, Uppsala University, Uppsala, Sweden; 2 Laboratory of Molecular Medicine, Department of Clinical Immunology, Section 7631, University Hospital of Copenhagen, Rigshospitalet, Copenhagen, Denmark; 3 National Bioinformatics Infrastructure Sweden, Department of Biochemistry and Biophysics, Stockholm University, Science for Life Laboratory, Stockholm, Sweden; 4 Department of Medical Sciences, Rheumatology, Uppsala University, Uppsala, Sweden; 5 Rheumatology Unit, Department of Medicine Solna, Karolinska Institutet, Karolinska University Hospital, Stockholm, Sweden; 6 Academic Specialist Center, Center for Rheumatology, Stockholm, Sweden; 7 Barbara Volcker Center for Women and Rheumatic Diseases, Hospital for Special Surgery, New York City, New York, USA; 8 Faculty of Medicine, School of Health Sciences, University of Iceland, Reykjavík, Iceland; 9 Department of Medicine, Karolinska Institute Clinical Epidemiology Division, Stockholm, Sweden

**Keywords:** Lupus Erythematosus, Systemic, Autoantibodies, Inflammation

## Abstract

**Objective:**

SLE is a systemic autoimmune disease in which the complement system plays a key pathogenic role, yet the contribution of the lectin pathway remains unclear. Lectin pathway-dependent complement activation is initiated by pattern-recognition molecules complexed with mannose-binding lectin (MBL)-associated serine proteases (MASPs) and MBL-associated proteins (MAPs). Here, we combined biochemical and genetic analyses to explore associations between MASP/MAP proteins, SLE manifestations and autoantibody specificities.

**Methods:**

Serum concentrations of MASP-3, MAP-1 and MASP-2 were measured using ELISA in Swedish patients with SLE (n=522) and population-based matched controls (n=322). Serum type I interferon activity was measured by a cell-based reporter assay. Associations with SLE manifestations and autoantibodies were explored using logistic regression models. Single-nucleotide genetic variants spanning the *MASP1* and *MASP2* genes were analysed for associations with MASP/MAP levels and SLE manifestations.

**Results:**

Patients with MAP-1 serum concentrations in the highest quartile had significantly higher rates of discoid rash (OR 2.8 (95% CI 1.4 to 5.7)), haematological manifestations (OR 2.1 (95% CI 1.1 to 3.7)) and autoantibodies against Sm, RNP, SSA and SSB (ORs 2.4 (95% CI 1.3 to 4.6) to 3.6 (95% CI 1.7 to 7.7)). Patients in the highest quartiles of MAP-1 and MASP-2 had lower rates of anti-β2GP1 and anti-cardiolipin IgG and IgA anti-phospholipid antibodies (ORs 0.29 (95% CI 0.12 to 0.68) to 0.56 (95% CI 0.31 to 1.0)). Serum MAP-1 levels correlated with type I interferon activity (Spearman’s rho 0.34, p<0.0001), which mediated the associations of MAP-1 with haematological manifestations and Sm/RNP autoantibodies. Significant protein quantitative trait loci for MAP-1 and MASP-2 were identified; however, these did not show consistent associations with SLE or specific SLE manifestations.

**Conclusions:**

These results demonstrate a distinct clinical and serological SLE profile associated with components of the lectin pathway. The lectin pathway-regulatory protein MAP-1 displayed the strongest associations and may serve as a marker of SLE manifestations with a type I interferon signature.

**WHAT IS ALREADY KNOWN ON THIS TOPIC**

- Serum levels of components of the classical complement pathway associate with SLE disease activity and anti-dsDNA antibodies.
- In contrast, while the lectin pathway is a potent complement activation pathway, its role in SLE is poorly characterised.

**WHAT THIS STUDY ADDS**

- Circulating lectin pathway components associate with specific SLE manifestations in a manner that is non-overlapping with the classical complement pathway.
- The lectin pathway-regulatory protein mannose-binding lectin-associated protein-1 was identified as a marker of SLE manifestations with elevated type I interferon activity.

**HOW THIS STUDY MIGHT AFFECT RESEARCH, PRACTICE OR POLICY**

- Our study identifies a distinct clinical profile in patients with SLE with elevated lectin pathway activity, extending knowledge on the role of complement in SLE.

## Introduction

 SLE is a systemic autoimmune disease characterised by autoantibodies against nuclear antigens and disease manifestations in multiple organs.^1^ The complement system plays a key role in the pathogenesis of SLE. Consumption of serum complement serves as an indicator of active disease, and genetic deficiencies in C1q and other components of the classical complement pathway are strong risk factors for developing SLE.^2^ While most studies related to the complement system in patients with SLE have focused on the classical pathway, the lectin pathway represents a potent and independent complement activation pathway triggered by circulating pattern recognition molecules (PRMs).^3^ These include mannose-binding lectin (MBL) and three ficolins, of which ficolin-3 is present in the highest serum concentration and serves as the main initiator of the lectin pathway in humans.^4^ The lectin pathway PRMs form complexes with MBL-associated serine proteases (MASPs), which activate the complement cascade by cleaving complements C2, C4 and factor D, or with MBL-associated proteins (MAPs) that lack protease activity. In humans, five members of the MASP/MAP family are generated by alternative splicing of the *MASP1* and *MASP2* genes. *MASP1* gives rise to the serine proteases MASP-1 and MASP-3, and the truncated isoform MAP-1, which shares a common heavy chain with MASP-1 and MASP-3 but lacks a serine protease domain. In several studies, MAP-1 has been shown to inhibit complement activation.^5 6^ *MASP2* encodes the serine protease MASP-2 and a truncated isoform called small MAP (sMAP).

Genetic deficiencies in lectin pathway proteins do not appear to markedly increase susceptibility to SLE.^7^ However, a meta-analysis of *MBL2* haplotypes associated with low MBL serum levels indicated an association with SLE,^8^ and an acquired deficiency of the lectin pathway PRM ficolin-3 develops in a small subset of patients with SLE.^9^ In a recent study performed by our group, we further demonstrated that ficolin-3 is linked to cutaneous and haematological disease manifestations and correlates with autoantibody patterns in Swedish patients with SLE.^10^

Here, we investigated whether the MASP/MAP protein family exhibits a similar profile to ficolin-3 in SLE. We analysed serum levels of MASP-3, MAP-1 and MASP-2 in a large and well-characterised Swedish SLE cohort and explored associations with SLE manifestations and autoantibodies. We also aimed to identify genetic variants that regulate serum concentrations of the MASP proteins and determine if these are associated with SLE or SLE-related disease manifestations.

## Materials and methods

### Study participants

The Karolinska SLE cohort (Stockholm, Sweden) recruited patients and population-based controls between 2004 and 2014. Controls were individually matched to the first 322 patients with SLE for age, sex and region of residence. Patients with SLE fulfilled the classification criteria for SLE as defined by the American College of Rheumatology (ACR) and/or the Systemic Lupus International Collaborating Clinics.^11^,^13^ Clinical information was collected by a rheumatologist at the inclusion visit, when a medical file review was also performed to collect historical clinical data. Disease activity was assessed by the Systemic Lupus Activity Measure (SLAM) and the SLE Disease Activity Index (SLEDAI).^14 15^

### Complement, autoantibody and type I interferon activity measurements

Analyses were performed on samples collected at study inclusion. MASP-3, MAP-1 and MASP-2 levels were measured in serum using in-house ELISAs specific for the different MASP/MAP isoforms described in detail in previous publications.^16^,^18^ Due to sample constraints, measurements were available for 306 controls and 508 patients with SLE (MASP-3), 314 controls and 468 patients with SLE (MAP-1) or 322 controls and 519 patients with SLE (MASP-2). Analysis of ficolin-3 activity and complement components C1q, C4 and C3 was described previously.^10 19^ Autoantibodies were analysed using multiplex assays as previously described.^20^ Functional type I interferon activity was measured in serum using an in vitro assay using WISH reporter cells, as described previously.^21 22^ High type I interferon activity was defined as 2 SDs above the mean in controls.

### Genotyping

DNA samples from patients with SLE and controls were genotyped using the Illumina Global Screening Array. Standard quality control was performed with PLINK V.1.9,^23^ excluding samples with call rate <95%, heterozygosity or ancestry outliers (>5 SDs from the mean), relatedness or sex discrepancies and variants with call rate <98%, minor allele frequency <1% or Hardy-Weinberg equilibrium p<1e−4.^24^ Imputations were carried out on the Sanger Imputation Service.^25^ After imputation, variants with minor allele frequency <1% were excluded. Single nucleotide variants (SNVs) within the genomic coordinates of *MASP1* (chr3:186836148-chr3:187109508, approximately 273 kb) and *MASP2* (chr1:10987118-chr1:11206690, approximately 220 kb), based on human genome build GRCh37 and including a ±100 kb region flanking each gene, were then extracted from the cleaned dataset using PLINK.

### Statistical analysis

Patient and control characteristics are presented as medians with IQRs or percentages, depending on the data type. Groups were compared by non-parametric statistics (Mann-Whitney test) or χ^2^ tests. Correlations were assessed by Spearman’s rank correlation coefficient. ORs were calculated by logistic regression models adjusted for sex, age at follow-up and, in certain analyses type I interferon activity score. All p values were two-sided, and p<0.05 was considered statistically significant. All statistics and generation of graphs for clinical characteristics were performed using either SPSS (V.29.0.2.0) or GraphPad Prism (V.10.4.2). Bioinformatic data handling and statistical analyses were performed using R (V.4.3.23).

#### pQTL analysis

Protein quantitative trait locus (pQTL) analyses were performed to identify genetic variants associated with serum levels of MASP/MAP proteins. MASP-3, MAP-1 or MASP-2 serum concentrations were used as the quantitative variable, and their association with individual SNVs in the *MASP1* or *MASP2* gene regions were tested using linear regression with disease status as a covariate. Protein data were transformed by rank-based inverse normal transformation, cases and controls separately. Genotyped variants with a missing genotype rate greater than 10%, or deviation from Hardy-Weinberg equilibrium (p value<1e–10) were excluded. The genetic data handling was performed using PLINK (V.1.9).^23^ The Locuszoom plots were created using locuszoomr (V.0.3.5) package. Accounting for the correlation between genetic variants due to linkage disequilibrium (LD), resampling-based multiple testing correction was applied to control the family-wise error rate.^26^ SNVs with resampling-based adjusted p value<0.05 were considered significant. Stepwise multiple linear regression was used to test for independent pQTLs from the top SNV.^27^

#### Single nucleotide variant association with SLE

The association between SLE disease status and individual genetic variants was tested using the Cochran-Armitage trend test. To adjust for multiple testing, Westfall and Young’s resampling-based multiple testing correction was applied using PLINK (V.1.9).^23 28^

## Results

### Complement profiling reveals two complement clusters in SLE with distinct serological and clinical associations

**Figure 1:**
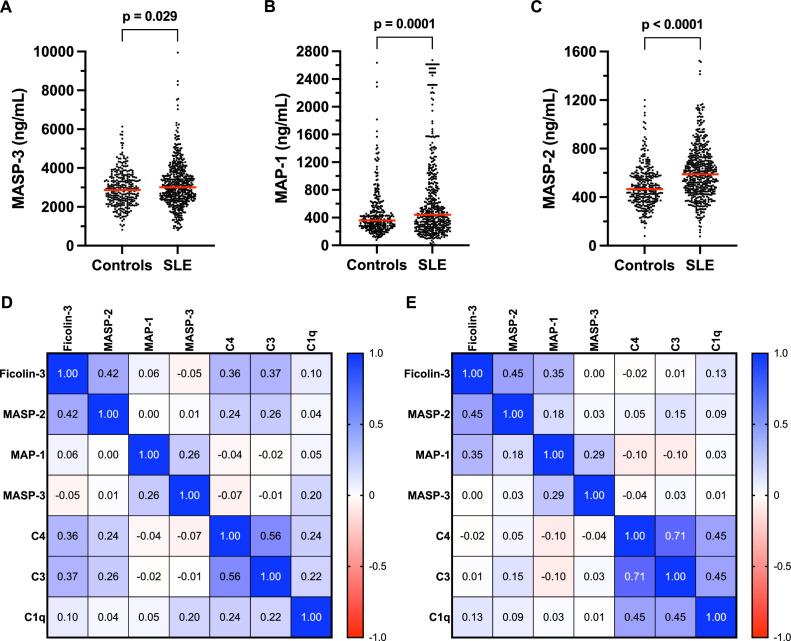
MASP protein family serum concentrations and correlation with complement factors in patients with SLE and matched controls. (**A-C**) MASP-3, MAP-1 and MASP-2 concentrations were measured in serum from Swedish patients with SLE and matched population controls using in-house ELISAs. (**A**) MASP-3 concentration in serum was 3003 ng/mL (2404–3710) for patients with SLE (n=508) and 2867 ng/mL (2306–3503) for controls (n=306), p=0.029. (**B**) MAP-1 concentration in serum was 441 ng/mL (265–845) for patients with SLE (n=468) and 359 ng/mL (267-513) for controls (n=314), p=0.0001. (**C**) MASP-2 concentration in serum was 589 ng/mL (451–727) for patients with SLE (n=519) and 466 ng/mL (373–579) for controls (n=322), p<0.0001. Data are presented as medians with IQR. Red horizontal lines indicate the median values. Statistical analysis was performed with the Mann-Whitney U test. (**D-E**) Correlation matrices showing Spearman’s rank correlation coefficients for ficolin-3 activity and MASP-2, MAP-1, MASP-3, C4, C3 and C1q serum concentrations in controls (**D**) and patients with SLE (**E**). The colour key indicates the degree and direction of correlation. P values for the correlation coefficients are shown in online supplemental table 14. MAP, mannose-binding lectin-associated protein; MASP, mannose-binding lectin-associated serine protease.

The median age in the cohort was 47 (34–58) years for patients with SLE and 48 (35–59) years for controls, with a median disease duration of 9 years (2–20) for the patients with SLE. The gender distribution was 85% females in patients with SLE and slightly higher in controls (92%). Serum concentrations of MASP-3, MAP-1 and MASP-2 were significantly higher in patients with SLE compared with controls, although all three proteins displayed a wide range with low or undetectable levels in some subjects (figure 1A–C). One patient with SLE had undetectable levels of MASP-3 in serum, and one individual in the control group had undetectable levels of MASP-2. Additionally, two patients with SLE had less than 10 ng/mL of MAP-1 in their serum. Interestingly, one of these patients, identified in our previous study, was also found to have very low levels of ficolin-3.^10^

To explore correlations between lectin pathway components and other complement factors, we combined the MASP-3, MAP-1 and MASP-2 measurements with our previously published data on serum ficolin-3 activity,^10^ and C1q, C4 and C3 plasma concentrations which were previously analysed in this cohort (figure 1D,E).^19^ In controls, there were moderate correlations among most complement factors, with the exception of MAP-1 and MASP-3, while in patients with SLE, two distinct clusters became apparent. As expected, C1q, C4 and C3 all showed a strong correlation and formed a classical pathway cluster, while ficolin-3, MASP-2 and MAP-1 showed a moderate or strong degree of correlation with each other. Notably, lectin pathway components displayed no correlation with classical pathway components, suggesting that these two pathways are regulated independently in patients with SLE.

Next, we compared MASP-3, MAP-1 and MASP-2 levels in patients with or without specific SLE manifestations or autoantibody specificities. MAP-1 serum concentrations were significantly higher in patients with discoid rash, haematological manifestations (leucopenia and lymphopenia), or those who fulfilled the anti-Sm criterion. In the autoantibody analysis, MAP-1 levels were markedly higher in patients with either Sm, Sm/RNP or SSA/SSB antibodies, while they were significantly lower in patients with anti-phospholipid antibodies (aPLs) (online supplemental table 2). These results revealed a pattern of associations that was nearly identical to our previous analysis of ficolin-3 in this cohort. MASP-2 displayed similar but less pronounced associations with autoantibodies, notably characterised by significantly lower MASP-2 serum levels in aPL-positive patients, whereas associations with ACR criteria manifestations were not significant (online supplemental table 3). In line with the absence of correlation with other lectin pathway components, serum levels of MASP-3 did not show any of the above associations. MASP-3 serum levels were slightly lower in patients with malar rash, serositis and nephritis (online supplemental table 1). Serum MASP-3 or MASP-2 did not correlate with SLE disease activity measured by SLAM or SLEDAI scores, whereas serum MAP-1 showed a weak positive correlation with both SLAM (r=0.17, p=0.0073) and SLEDAI (r=0.12, p=0.011). Furthermore, ongoing treatment with immunosuppressants did not correlate with levels of MASP-3 and MAP-1, whereas patients with ongoing treatment with antimalarials had lower MASP-2 serum levels (online supplemental tables 1–3).

**Figure 2:**
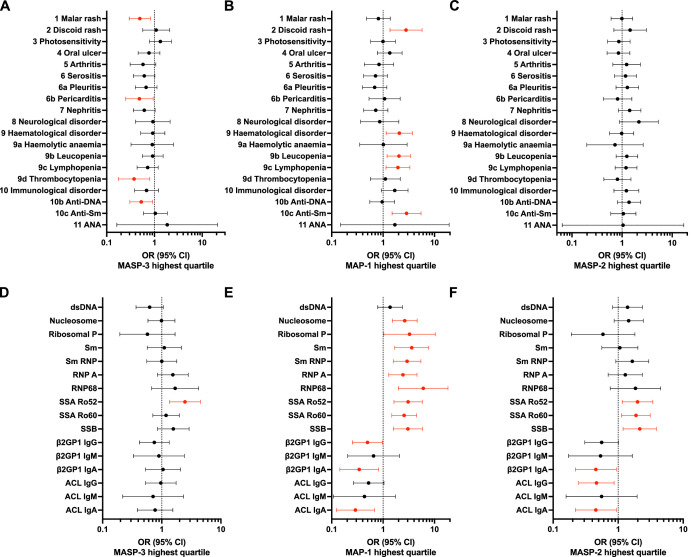
Associations between MASP-3, MAP-1 and MASP-2 with SLE manifestations and autoantibodies in patients with SLE. The graphs show forest plots with ORs and 95% CIs for patients with serum concentrations of MASP-3, MAP-1 or MASP-2 in the highest quartile (Q4) compared with the lowest quartile (Q1) of each respective dataset. All analyses were adjusted for sex and age at follow-up. Significant ORs (p<0.05, unadjusted for multiple comparisons) are shown in red. (**A-C**) ORs for SLE criteria according to the 1982 ACR classification for (**A**) MASP-3 (n=498–502), (**B**) MAP-1 (n=458–462) and (**C**) MASP-2 (n=509–513). (**D-E**) ORs for autoantibody specificities for (**D**) MASP-3 (n=487–504), (**E**) MAP-1 (n=447–464) and (**F**) MASP-2 (n=497–515). ACL, anti-cardiolipin; ACR, American College of Rheumatology; MAP, mannose-binding lectin-associated protein; MASP, mannose-binding lectin-associated serine protease.

In a logistic regression model adjusted for sex and age at follow-up, the associations for MAP-1 and MASP-2 were confirmed. Patients in the highest MAP-1 quartile had significantly higher rates of discoid rash (OR 2.8 (95% CI 1.4 to 5.7)), leucopenia (OR 2.0 (95% CI 1.2 to 3.5)), lymphopenia (OR 1.9 (95% CI 1.1 to 3.3)) and anti-Sm autoantibodies (OR 2.9 (95% CI 1.5 to 5.5)) (figure 2, online supplemental tables 4–6). Notably, both MAP-1 and MASP-2 showed an inverse relationship with aPLs, specifically anti-β2GP1 and anti-cardiolipin antibodies of the IgG and IgA isotypes (ORs for MAP-1 0.29 (95% CI 0.12 to 0.68) to 0.52 (95% CI 0.26 to 1.0); ORs for MASP-2 0.45 (95% CI 0.22 to 0.94) to 0.56 (95% CI 0.31 to 1.0)). Taken together, MAP-1 and, to some extent, MASP-2 replicated serological and clinical associations previously observed for ficolin-3.^10^

As the correlation analysis indicated distinct clusters of classical and lectin pathway proteins in SLE, we explored the clinical associations for C4, C3 and C1q. In the logistic regression model comparing the highest quartile with the lowest, C4 and C3 showed an inverse association with haematological manifestations (OR C4 0.36 (95% CI 0.19 to 0.65); C3 0.53 (95% CI 0.30 to 0.94)), while low C3 was also associated with nephritis (OR 0.42 (95% CI 0.25 to 0.71). As expected, C1q, C4 and C3 levels were markedly suppressed in patients with anti-dsDNA or anti-nucleosome antibodies (ORs 0.10 (95% CI 0.05 to 0.18) to 0.18 (95% CI 0.10 to 0.32)), while C4 and C3 were also significantly lower in patients with aPLs (ORs for C4 0.13 (95% CI 0.03 to 0.58) to 0.25 (95% CI 0.08 to 0.78); C3 0.18 (95% CI 0.04 to 0.86) to 0.35 (95% CI 0.18 to 0.68)) (online supplemental figure 1, online supplemental tables 7–9).

### Clinical and serological associations with serum MAP-1 are in part mediated by elevated type I interferon activity

**Figure 3:**
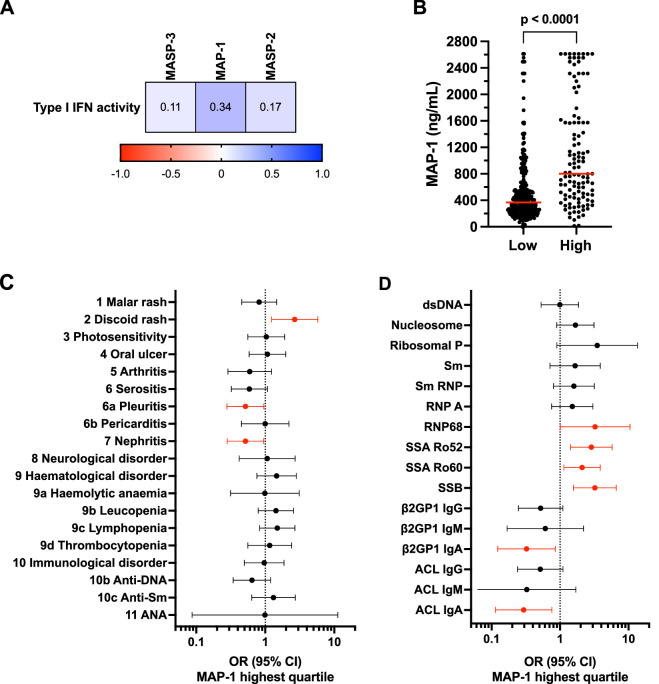
Type I interferon activity correlates with MAP-1 levels and mediates the association with haematological manifestations. (**A**) Correlation matrices showing Spearman’s rank correlation coefficients for type I interferon activity score with MASP-3 (⍴=0.11, p=0.024), MAP-1 (⍴=0.34, p<0.0001), MASP-2 (⍴=0.17, p=0.00036). The colour key indicates the degree and direction of correlation. (**B**) MAP-1 concentration in patients with high type I interferon activity compared with patients with low type I interferon activity. High type I interferon activity was defined as 2 SDs above the mean in controls. MAP-1 concentration in serum was 801 ng/mL (455–1574) for patients with high type I interferon activity (n=115) and 367 ng/mL (231–563) for patients with low activity (n=297), p<0.0001. Data are presented as medians with IQR. Red horizontal lines in the graph indicate the median values. Statistical analysis was performed with the Mann-Whitney U test. (**C-D**) The graphs show forest plots with ORs and 95% CIs for patients with MAP-1 serum concentrations in the highest quartile (Q4) compared with the lowest quartile (Q1). All analyses were adjusted for sex, age at follow-up and high type I interferon activity. High type I interferon activity was defined as 2 SDs above the mean in controls. Significant ORs (p<0.05, unadjusted for multiple comparisons) are shown in red. (**C**) ORs for SLE criteria according to the 1982 ACR classification (n=410–412). (**D**) ORs for autoantibody specificities (n=410–412). ACL, anti-cardiolipin; ACR, American College of Rheumatology; IFN, interferon; MAP, mannose-binding lectin-associated protein; MASP, mannose-binding lectin-associated serine proteases.

MAP-1 showed an association with SLE manifestations that are typically enriched in patients with an elevated type I interferon signature, such as haematological manifestations and anti-Sm/RNP antibodies.^29 30^ We explored the correlation between lectin pathway components and serum type I interferon activity measured by a cell-based reporter assay. MAP-1 displayed the strongest correlation, and patients with high type I interferon activity had markedly elevated MAP-1 serum levels, indicating that MAP-1 is an interferon-regulated circulating protein (figure 3A,B).

Next, we adjusted the MAP-1 logistic regression model for type I interferon activity using a previously described binning strategy,^31^ which attenuated the associations with leucopenia (OR 1.4 (95% CI 0.79 to 2.6)), lymphopenia (OR 1.5 (95% CI 0.83 to 2.7)) and anti-Sm autoantibodies (OR 1.3 (95% CI 0.64 to 2.7)), indicating that the link between MAP-1 and these SLE features are mediated by a type I interferon response. In contrast, associations with discoid rash (OR 2.7 (95% CI 1.2 to 5.8)) as well as the negative associations with IgG/IgA aPLs (ORs 0.29 (95% CI 0.11 to 0.76) to 0.52 (95% CI 0.25 to 1.1)) largely remained after adjustment (figure 3C,D, online supplemental table 5).

### pQTL analysis identifies genetic determinants of circulating MASP levels

These results indicated that a shared and partially type I interferon-dependent mechanism regulates serum levels of lectin pathway proteins in SLE. Nonetheless, genetic polymorphisms are known to influence serum levels of complement proteins. To gauge the genetic contribution to the association of MASP/MAP serum levels with SLE manifestations, we performed cis-pQTL analysis exploring the associations between MAP-1, MASP-3 and MASP-2 serum levels and SNVs in the corresponding *MASP1* and *MASP2* gene regions.

**Figure 4:**
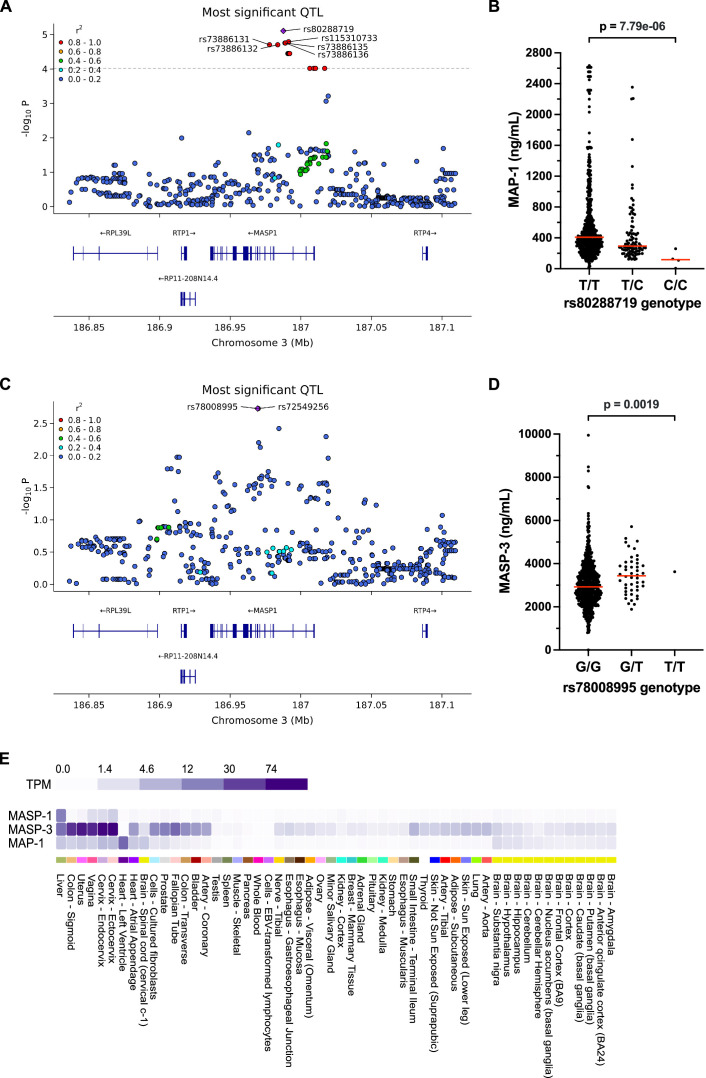
Genetic associations with MAP-1 and MASP-3 concentrations in the *MASP1* gene region. (**A**) SNVs in the *MASP1* gene region were tested for their association with MAP-1 concentration in patients with SLE and controls combined (n=681). The locus zoom plot shows unadjusted p values for individual SNVs. The dotted line indicates the significance threshold (adjusted p<0.05). *r*^2^ indicates the Pearson correlation coefficient of allele count with the top SNV, indicated with a diamond shape. (**B**) Effects of top SNV rs80288719 on MAP-1 levels in serum. MAP-1 levels were 408 (277–660) ng/mL for the T/T genotype (n=581), 293 (225–464) ng/mL for the T/C genotype (n=95) and 117 (32–225) ng/mL for the C/C genotype (n=4), p value unadjusted 7.79e−06, adjusted 0.0012. Data are presented as median with IQR. Red horizontal lines in the graph indicate median values. (**C**) SNVs in the *MASP1* gene region were tested for their association with MASP-3 concentration in the combined data set (n=709). The locus zoom plots show unadjusted p values for individual SNVs. There were no SNVs above the significance threshold (adjusted p<0.05). *r*^2^ indicates the Pearson correlation coefficient of allele count with the top SNV, indicated with a diamond shape. (**D**) Effect of the rs78008995 genotype on MASP-3 levels in serum. MASP-3 levels were 2927 (2355–3592) ng/mL for the G/G genotype (n=650), 3438 (2771–4032) ng/mL for the G/T genotype (n=46) and 3623 ng/mL for the T/T genotype (n=1), p value unadjusted 0.0019, adjusted 0.17. Data are presented as median with IQR. Red horizontal lines in the graph indicate median values. (**E**) Tissue expression of *MASP1* isoforms in human tissues. MASP-1 is predominantly expressed in the liver, whereas MASP-3 is expressed in a larger subset of tissues and MAP-1 is primarily expressed in the left ventricle of the heart. Data are presented as TPM, a normalised method to estimate gene expression levels adjusted for transcript length and sequencing depth. The figure was generated using publicly available data from the GTEx portal release V.10. GTEx, Genotype-Tissue Expression; MAP, mannose-binding lectin-associated protein; MASP, mannose-binding lectin-associated serine protease; QTL, quantitative trait locus; SNV, single nucleotide variant; TPM, transcripts per million.

17 SNVs in a 39 kB region spanning exons 1–4 of the *MASP1* gene were significantly associated with serum MAP-1 concentrations (figure 4A,B, analysis stratified for disease status is shown in online supplemental figure 2). Significant SNVs were highly correlated, and analysis using LDlink confirmed a high degree of LD (*r*^2^>0.9) in European populations.^32^ The top hit for MAP-1, rs80288719, was in high LD (*r*^2^=0.98) with a previously reported SNV, rs7625133, associated with low levels of MAP-1 in plasma in healthy subjects.^33^ rs80288719 was a specific pQTL for MAP-1, showing no association with MASP-3 (p value unadjusted 0.35, adjusted p=1.0).

No SNVs in the *MASP1* gene region were significantly associated with MASP-3 serum levels after multiple testing correction; however, two intronic SNVs in high LD were associated with higher MASP-3 levels with borderline significance (top hit rs78008995) (figure 4C,D, analysis stratified for disease status is shown in online supplemental figure 3). rs78008995 did not have a strong effect on MAP-1 serum levels (p value unadjusted 0.085, adjusted p=1.0).

The primary *MASP1* transcript is spliced into three protein-coding transcripts, which are translated into MASP-1, MASP-3 and MAP-1. We analysed Genotype-Tissue Expression (GTEx) portal data to investigate if significant pQTLs were associated with altered *MASP1* expression.^34^ The MAP-1 pQTL rs80288719 was associated with decreased *MASP1* expression in GTEx (table 1). Interestingly, the strongest effect was seen in the heart, where MAP-1 is the sole *MASP1* isoform to be expressed (figure 4E), in agreement with a selective effect on serum MAP-1 levels of this pQTL. In contrast to the restricted expression of the MAP-1 transcript, GTEx showed a broad expression of MASP-3 in a range of tissues. The MASP-3 pQTL rs78008995 was associated with higher *MASP1* transcription in tissues with expression of MASP-3 but not MASP-1 or MAP-1 (table 1).

**Table 1:** Significant tissue eQTLs in GTEx portal for the pQTLs identified in the study

Gene	SNP ID	Tissue	NES	P value	TPM in tissue			
					MASP-1	MASP-3	MAP-1	MASP-2
*MASP1*	rs80288719	Heart – left ventricle	−0.36	1.3e−14	0.0200	0.00	18.2	n/a
*MASP1*	rs80288719	Nerve – tibial	−0.29	4.0e−7	0.0700	1.78	0.230	n/a
*MASP1*	rs80288719	Artery – aorta	−0.29	8.5e−6	0.0600	3.95	0.150	n/a
*MASP1*	rs80288719	Adrenal gland	−0.51	9.8e−6	0.100	0.890	0.105	n/a
*MASP1*	rs78008995	Adipose – subcutaneous	0.54	7.5e−10	0.110	3.15	0.150	n/a
*MASP1*	rs78008995	Cells – cultured fibroblasts	0.28	1.1e−5	0.150	9.95	1.17	n/a
*MASP1*	rs78008995	Oesophagus – muscularis	0.37	4.8e−5	0.0300	0.845	0.0700	n/a
*MASP1*	rs78008995	Thyroid	0.40	1.3e−4	0.120	2.51	0.170	n/a
*MASP2*	rs72550870	No significant eQTLs	n/a	n/a	n/a	n/a	n/a	n/a
*MASP2*	rs1033638	Nerve – tibial	0.18	4.1e−17	n/a	n/a	n/a	4.16
*MASP2*	rs1033638	Liver	−0.31	9.1e−7	n/a	n/a	n/a	25.8
*MASP2*	rs1033638	Adipose – subcutaneous	0.11	2.7e−6	n/a	n/a	n/a	1.92
*MASP2*	rs1033638	Artery – aorta	0.13	3.3e−5	n/a	n/a	n/a	1.68
*MASP2*	rs1033638	Thyroid	0.090	6.8e−5	n/a	n/a	n/a	2.00
*MASP2*	rs1033638	Skin – sun exposed (lower leg)	0.090	1.4e−4	n/a	n/a	n/a	3.16
*MASP2*	rs41307788	No significant eQTLs	n/a	n/a	n/a	n/a	n/a	n/a

Significant GTEx tissue eQTLs for the *MASP1* and *MASP2* genes, respectively, are shown along with expression levels of *MASP1* and *MASP2* transcripts. Data were obtained from the GTEx portal release V.10.

eQTL, expression quantitative trait locus; GTEx, Genotype-Tissue Expression; MAP, mannose-binding lectin-associated protein; MASP, mannose-binding lectin-associated serine protease; NES, normalised effect size; pQTL, protein quantitative trait locus; SNP, single nucleotide polymorphism; TPM, transcripts per million.

**Figure 5:**
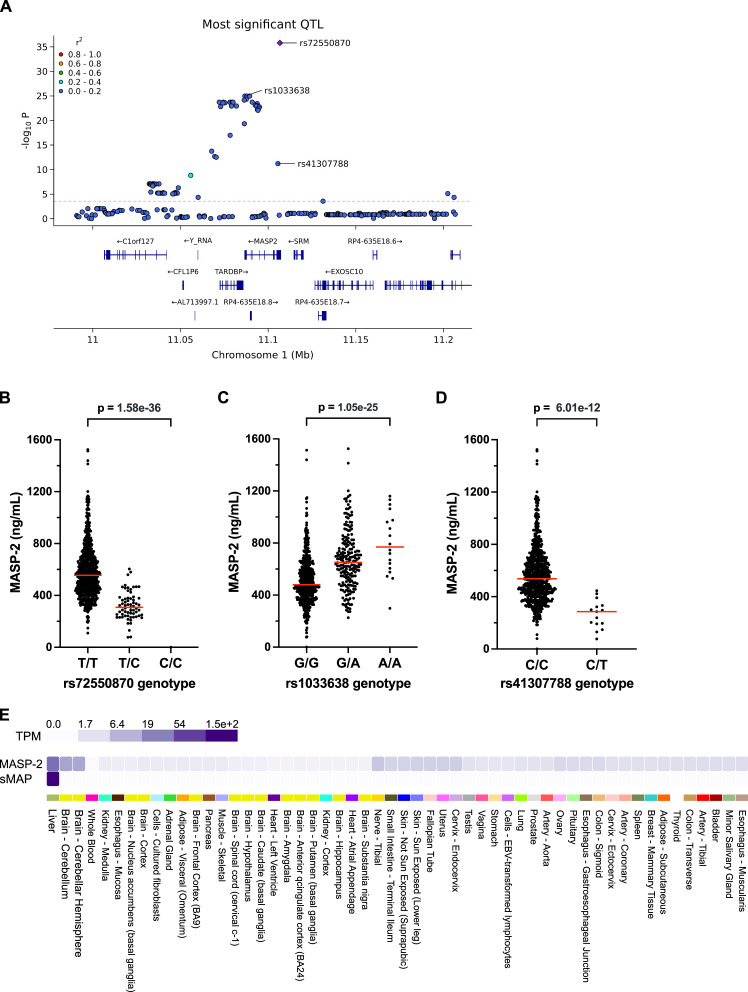
Genetic associations with MASP-2 concentration in the *MASP2* gene region. (**A**) SNVs in the *MASP2* gene region were tested for their association with MASP-2 concentration in patients with SLE and controls combined (n=735). The locus zoom plot shows unadjusted p values for individual SNVs. The dotted line indicates the significance threshold (adjusted p<0.05). *r*^2^ indicates the Pearson correlation coefficient of allele count with the top SNV, indicated with a diamond shape. (**B**) Effects of the first independent pQTL rs72550870 on MASP-2 levels in serum. MASP-2 levels were 555 (437–692) ng/mL for the T/T genotype (n=669), 308 (243–379) ng/mL for the T/C genotype (n=65) and 0 ng/mL for the C/C genotype (n=1), p value unadjusted 1.58e−36, adjusted <1e−04. Data are presented as medians with IQRs. Red horizontal lines in the graph indicate the median values. (**C**) Effects of the second independent pQTL rs1033638 on MASP-2 levels in serum. MASP-2 levels were 479 (382−605) ng/mL for the G/G genotype (n=515), 651 (512−793) ng/mL for the G/A genotype (n=201) and 769 (599−997) ng/mL for the A/A genotype (n=18), p value unadjusted 1.05e−25, adjusted <1e−04. (**D**) Effects of the third independent pQTL rs41307788 on MASP-2 levels in serum. MASP-2 levels were 538 (424−679) ng/mL for the C/C genotype (n=720), and 286 (192−333) ng/mL for the C/T genotype (n=15), p value unadjusted 6.01e−12, adjusted <1e−04. (**E**) Tissue expression of *MASP2* isoforms in human tissues. MASP-2 is primarily expressed in the liver and brain, whereas sMAP is expressed in the liver. Data is presented as TPM, a normalised method to estimate gene expression levels adjusted for transcript length and sequencing depth. The figure was generated using publicly available data from the GTEx portal release V.10. GTEx, Genotype-Tissue Expression; MASP, mannose-binding lectin-associated serine protease; QTL, quantitative trait locus; sMAP, small mannose-binding lectin-associated protein; SNV, single nucleotide variant; TPM, transcripts per million.

82 SNVs in the *MASP2* gene region were significantly associated with serum MASP-2 concentrations (figure 5A, analysis stratified for disease status is shown in online supplemental figure 4), and among these, stepwise linear regression identified three independent MASP-2 pQTLs. The top hit was rs72550870, a previously described asparagine to glycine (p.D120G) substitution in the MASP-2 heavy chain, causing complete MASP-2 deficiency in the homozygous state.^35^ In our dataset, the presence of the rs72550870 minor allele was associated with an approximately 50% reduction in MASP-2 serum levels (figure 5B). One control individual was homozygous for the rs72550870 alternative allele and had no detectable MASP-2 in serum.

Two additional pQTLs independent of rs72550870 were identified. rs1033638, located in the *MASP2* 3’ untranslated region, was associated with high MASP-2 levels (figure 5C) and was in high LD with several significant SNVs spanning exons 8–11 of the *MASP2* gene and the downstream *TARDBP* gene, a region that includes a previously reported SNV linked to elevated MASP-2 levels.^36^ A previously undescribed missense variant, rs41307788, causing a cysteine-to-tyrosine substitution (p.C156Y) in the MASP-2 heavy chain, was associated with low serum MASP-2 independent of the two top pQTLs (figure 5D).

GTEx confirmed MASP-2 to be predominantly expressed in the liver (figure 5E). The rs72550870 and rs41307788 missense variants had no significant effects on *MASP2* messenger RNA levels, suggesting a post-transcriptional effect on circulating MASP-2. In contrast, the non-coding pQTL rs1033638 was associated with *MASP2* expression in a number of tissues, in agreement with an effect on *MASP2* transcription by the functional variant tagged by this SNV (table 1).

### Genetic associations with disease status and SLE manifestations

Lastly, we explored if the significant MAP-1 and MASP-2 pQTLs colocalised with genetic signals for SLE-related traits. The MAP-1 pQTL rs80288719 was not associated with SLE diagnosis (p value unadjusted 0.71, adjusted 1.0). We also calculated ORs for SLE manifestations and autoantibody specificities for carriers of the rs80288719 alternative allele, which revealed a protective effect against leucopenia (OR 0.54 (95% CI 0.31 to 0.95)). Still, no consistent associations were found that could explain the pattern of clinical and autoantibody associations observed for serum MAP-1 (online supplemental table 10). Likewise, MASP-2 loss-of-function variants were not associated with SLE (rs72550870 p value unadjusted 0.87, adjusted 1.0; rs41307788 p value unadjusted 0.80, adjusted 1.0). As for the MAP-1 pQTLs, genetically predicted MASP-2 did not replicate the autoantibody associations observed for serum MASP-2. However, it could be noted that MASP-2 haploinsufficiency was associated with protection from nephritis in this cohort, both when analysing rs72550870 separately (OR 0.36 (95% CI 0.16 to 0.81)) and when combining the two missense variants rs72550870 and rs41307788 (OR 0.45 (95% CI 0.22 to 0.92)) (online supplemental tables 11–13).

## Discussion

Although altered serum complement levels in SLE were recognised as early as the 1950s,^37^ the role of the lectin pathway in SLE has not been conclusively resolved. Here, we explored serum concentrations of MASP/MAP proteins in a large and well-characterised Swedish SLE cohort and show that components of the lectin pathway display a distinct and reproducible association with specific SLE manifestations and autoantibody specificities.

Serum concentrations of all three MASP/MAPs were elevated in patients with SLE compared with controls, consistent with previous studies.^38^,^40^ Our correlation analysis revealed two separate complement clusters in patients with SLE, where ficolin-3, MAP-1 and MASP-2 formed a lectin pathway cluster that displayed little or no correlation with classical pathway components. Consistent with these observations, we identified distinct serological and clinical associations with serum levels of classical and lectin pathway proteins. We observed elevated MAP-1 and MASP-2 concentrations in patients positive for the main SLE autoantibodies, such as dsDNA/nucleosomes/RNP and SSA/SSB specificities, whereas serum concentrations were suppressed in patients with aPLs. Notably, these results were in agreement with our previous observations for the lectin pathway PRM ficolin-3 in two Swedish SLE cohorts.^10^ Furthermore, ficolin-3 activity was associated with discoid rash and haematological manifestations, a pattern identical to the MAP-1 associations in the present study.

These observations point to a common mechanism that determines the circulating concentrations of lectin pathway proteins in SLE. To put our results into context, a distinct pattern of associations was observed for components of the classical pathway. Autoantibody-containing immune complexes activate complement through C1q and the classical pathway in SLE.^2^ If the activation is sufficiently strong to overwhelm the synthesis rate, it depletes circulating complement factors, resulting in low serum complement, which is a common observation during SLE disease flares and in patients with nephritis, haematological manifestations or dsDNA antibodies.^19 41^ Indeed, these well-known features in SLE were also evident in the present cohort.

What explains the pattern observed for serum lectin pathway components in relation to disease manifestations in SLE? We identified MAP-1 as a potential novel interferon-regulated protein, and patients classified as having high type I interferon activity had markedly elevated circulating MAP-1. Activation of the type I interferon system occurs physiologically during viral infections but is also closely linked to the pathogenesis of SLE.^42 43^ Patients with SLE with an elevated interferon signature show a distinct pattern of signs and symptoms, typically with a high rate of haematological manifestations and SLE-associated autoantibodies against nuclear antigens such as anti-Sm. Adjustment for type I interferon activity attenuated the associations between MAP-1 and these manifestations, indicating that they are mediated by a type I interferon response that upregulates MAP-1 serum levels. MAP-1 has no protease activity but can compete with the lectin pathway proteases for binding to ficolin-3 and may therefore function as a competitive lectin pathway inhibitor.^6^ We show that MAP-1 is strongly upregulated by type I interferons, which could indicate a need for suppression of excessive lectin pathway activation during prolonged inflammation, or alternatively, a distinct MAP-1 function in the antiviral response that has yet to be revealed.

Furthermore, the presence of elevated MAP-1, MASP-2 and ficolin-3 serum levels in patients with Sm/RNP and SSA/SSB autoantibodies also indicates that the lectin pathway is not activated in a manner that depletes circulating components in these SLE subgroups. An exception from this pattern was the inverse association with aPLs, which was observed for both MAP-1 and MASP-2, as well as for ficolin-3 in our previous study. These associations were independent of serum type I interferon activity, and aPL status in SLE does not appear to be a strongly interferon-regulated trait.^44^ Instead, as low complement levels in SLE may be evidence of complement activation, it is tempting to speculate that lectin pathway activation and turnover in aPL-positive subjects cause a relative depletion of lectin pathway components in this serological subtype. However, the potential mechanism of lectin pathway activation in this subset of patients with SLE so far remains hypothetical.

MASP-3 appeared uncoupled from other components of the lectin pathway. MASP-3 has a distinct function serving as an activator of factor D and the alternative complement pathway, but is dispensable for lectin pathway activation in both rodents and humans.^45 46^ Its turnover in serum may therefore be influenced by the alternative pathway rather than lectin pathway activation. It is also possible that its broad expression in a range of tissues provides redundant mechanisms to maintain constant MASP-3 levels in the circulation.

In the genetic analysis, we replicated previously reported pQTLs for serum levels of MAP-1 and MASP-2, and identified a novel *MASP2* loss-of-function variant (rs41307788) with an allele frequency of approximately 0.01 in European populations. However, while serum MAP-1 and MASP-2 displayed strong associations with clinical manifestations and several autoantibody specificities, none of the pQTLs for MAP-1 and MASP-2 colocalised with a consistent genetic signal for these SLE traits. Therefore, the associations between MASP/MAPs and SLE manifestations do not appear to be explained by genetic causes, but rather a consequence of altered turnover of circulating proteins, such as a response to interferons or the postulated consumption in patients with aPL-positive SLE.

Nonetheless, our results provide a comprehensive survey of the genetic regulation of lectin pathway proteins in SLE. While MAP-1 and MASP-3 both are transcribed from the *MASP1* gene, GTEx data showed a non-overlapping expression pattern and the pQTL analysis identified no shared strong pQTLs, indicating that independent mechanisms regulate the expression of these two splice products. Our analyses also showed that, in contrast to MASP-2 and many other complement proteins, extrahepatic tissues are the main sources of circulating MAP-1 and MASP-3. We identified tissue-specific pQTLs that linked low MAP-1 serum levels to decreased *MASP1* gene expression in heart tissue, and thus confirmed previous observations that MAP-1 is mainly expressed in the heart.^5 6^

The top MASP-2 pQTL was a previously described missense variant causing a p.D120G substitution in the CUB1 domain of the MASP-2 heavy chain. The mutation renders MASP-2 incapable of associating with MBL and presumably also other circulating lectin pathway PRMs.^35 47^ It has been shown that association with a PRM retains the MASPs in the circulation and prolongs their half-life,^48^ and an inability to form complexes with PRMs is therefore compatible with absent or very low levels of circulating MASP-2.

We also identified a p.C156Y substitution in the MASP-2 heavy chain that was independently associated with low serum MASP-2 levels. It disrupts a predicted disulfide bond in the MASP-2 EGF domain, which, similarly to the CUB1 domain, is involved in MASP-2 dimerisation and PRM association.^49^ A 4-amino acid in-frame insertion present in East Asian populations (p.156-159dupCHNH, rs777682380) affects the same protein region and was reported to abolish the binding of MASP-2 to MBL.^47^ It, therefore, appears likely that the p.C156Y variant, like p.D120G, leads to low circulating MASP-2 levels due to an inability to form complexes with PRMs. However, as the exact epitopes of the monoclonal MASP-2 antibodies in our ELISA assay are not known, it also remains a theoretical possibility that the p.C156Y MASP-2 variant is not recognised by the assay.

MASP-2 haploinsufficiency was not associated with SLE; instead, we observed one control individual homozygous for rs72550870 without detectable MASP-2 in their serum. Although initially considered a monogenic immunodeficiency, a number of apparently healthy MASP-2-deficient subjects have been identified incidentally in genetic studies, and the penetrance of an immunodeficient phenotype in MASP-2-deficient subjects appears to be low.^7^ It could be noted that MASP-2 haploinsufficiency was associated with protection from nephritis in our cohort, which is especially noteworthy as an MASP-2 inhibitory antibody is in clinical trials for nephritis-related conditions.^50^ However, our analysis was exploratory and needs to be further replicated in an independent cohort.

A limitation of the study is that assays for MASP-1 and sMAP were not available; therefore, our study does not provide a complete survey of all MASP/MAP isoforms in humans. For future studies, it would be interesting to explore if MASP-1 and sMAP follow the same pattern as MASP-2 and MAP-1 in SLE. Another limitation is that patients and controls in the study were mostly of Northern European origin; therefore, our study does not allow for conclusions about the lectin pathway in patients with SLE of other ancestries.

## Conclusions

In conclusion, we demonstrate a distinct lectin pathway profile in SLE, where serological subtype strongly influences levels of complement proteins, but in a distinct and completely non-overlapping manner for the classical and lectin pathways. Furthermore, MAP-1 may serve as a marker of SLE manifestations linked to high type I interferon activity.

## Supplementary material

10.1136/lupus-2025-001890 online supplemental file 1

## Data Availability

Data are available upon reasonable request.
