# Supplementary material for: Lectin pathway of complement in SLE: MAP-1 as a marker of haematological manifestations and elevated type I interferon activity
Source: Lupus Sci Med. 2026 Apr 9;13(1):e001890. doi: 10.1136/lupus-2025-001890 (PMC13084892; doi:10.1136/lupus-2025-001890)
Supplement: online supplemental file 1 [file lupus-13-1-s001.pdf]

SUPPLEMENTARY MATERIAL

The Lectin Pathway of Complement in Systemic Lupus Erythematosus: MAP-1 as a Marker of Haematological Manifestations and Elevated Type I Interferon Activity

Linnea Lindelöf[1], Peter Garred[2], Mun-Gwan Hong[3], Sasha Wahl Vælum[2], Lotte Holten Petersen[2], Dag Leonard[4], Ahmed Sayadi[4], Vilija Oke[5,6], Timothy B. Niewold[7], Lina-Marcela Diaz-Gallo[5], Saedis Saevarsdottir[8,9], Iva Gunnarsson[5], Elisabet Svenungsson[5], Oskar Eriksson[1]*

Affiliations:

[1] Department of Immunology, Genetics and Pathology, Uppsala University, Uppsala, Sweden.

[2] Laboratory of Molecular Medicine, Department of Clinical Immunology, Section 7631, Rigshospitalet, University Hospital of Copenhagen, Copenhagen, Denmark.

[3] National Bioinformatics Infrastructure Sweden, Science for Life Laboratory, Department of Biochemistry and Biophysics, Stockholm University, Stockholm, Sweden.

[4] Department of Medical Sciences, Rheumatology, Uppsala University, Uppsala, Sweden.

[5] Division of Rheumatology, Department of Medicine, Solna, Karolinska Institutet, Karolinska University Hospital, Stockholm, Sweden.

[6] Center for Rheumatology, Academic Specialist Center, Stockholm Region, Stockholm.

[7] Hospital for Special Surgery, Barbara Volcker Center for Women and Rheumatic Diseases, New York, NY, USA.

[8] Faculty of Medicine, School of Health Sciences, University of Iceland, Reykjavík, Iceland.

[9] Division of Clinical Epidemiology, Department of Medicine, Solna, Karolinska Institutet, Karolinska University Hospital, Stockholm, Sweden.

* Corresponding author:

Address: The Rudbeck Laboratory, Uppsala University, S-751 85 Uppsala, Sweden

E-mail: oskar.eriksson@igp.uu.se

Phone: +46186175392

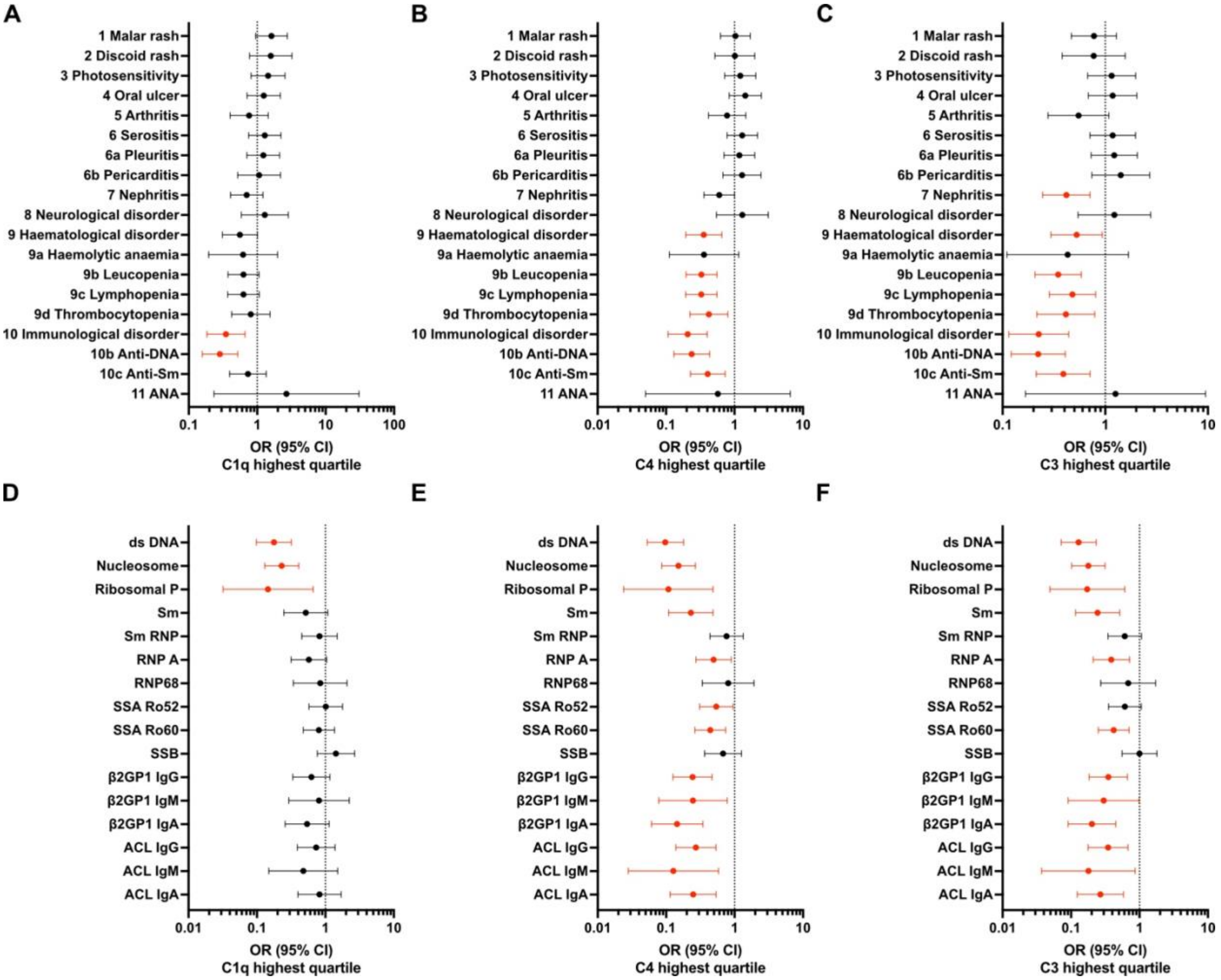

Supplementary Figure 1. Associations between C1q, C4, and C3 with SLE manifestations and autoantibodies in patients with SLE.

The graphs show forest plots with odds ratios (OR) and 95 % confidence intervals (CI) for patients with serum concentrations of C1q, C4, or C3, in the highest quartile (Q4) compared to the lowest quartile (Q1) of each respective data set. All analyses were adjusted for sex and age at follow-up. Significant ORs ($p<0.05$, unadjusted for multiple comparisons) are shown in red.

A-C. ORs for SLE criteria according to the 1982 ACR classification for (**A**) C1q (n=475-478), (**B**) C4 (n=501-506), and (**C**) C3 (n=501-506).

D-F. ORs for autoantibody specificities for (**D**) C1q (n=477-478), (**E**) C4 (n=489-508), and (**F**) C3 (n=489-508).

ACL: Anti-cardiolipin, ACR: American College of Rheumatology, ANA: Antinuclear antibody, dsDNA: double-stranded DNA, RNP: Ribonucleoprotein, Sm: Smith antigen, SSA/B: Sjögren's syndrome antigen A/B.

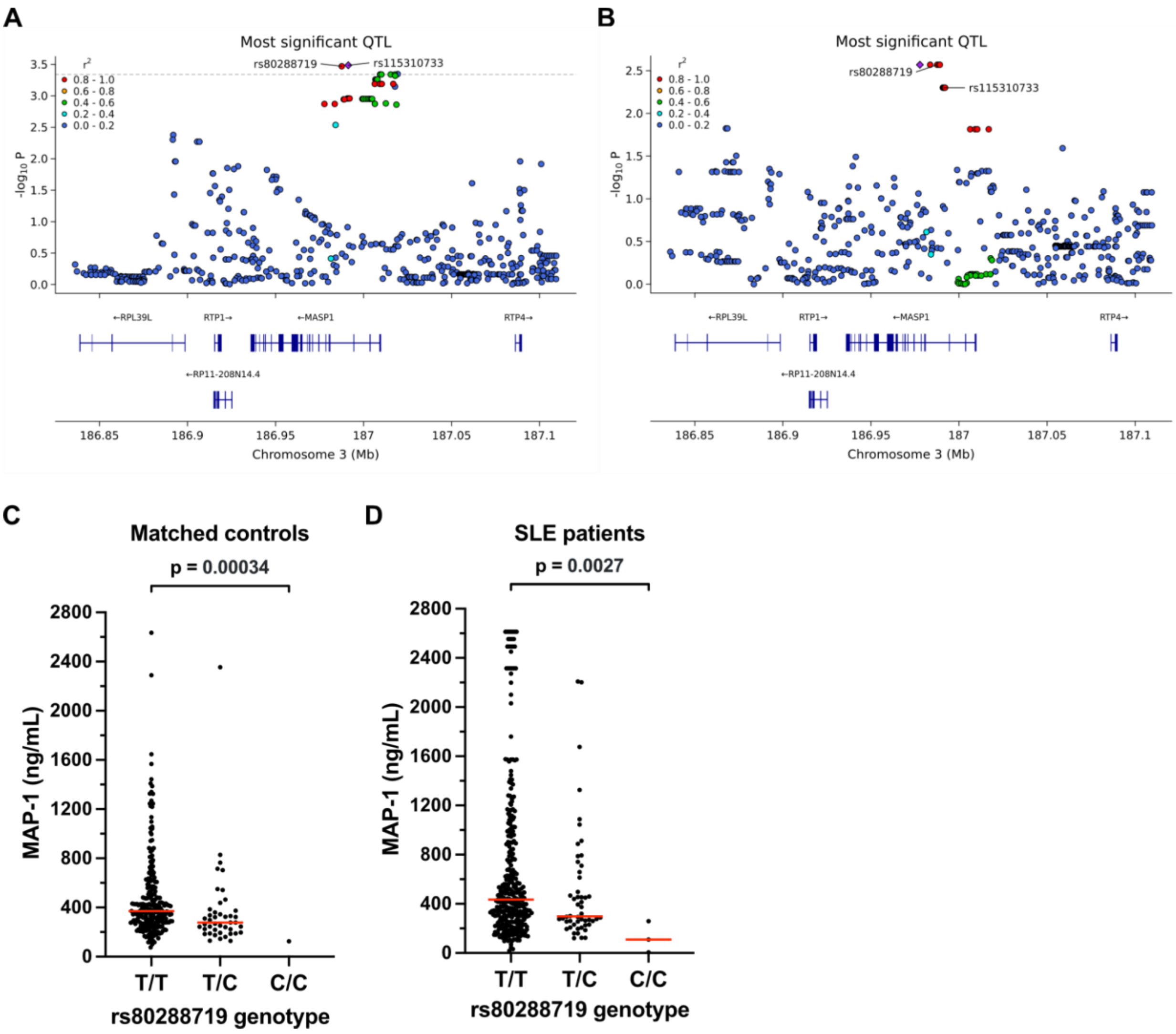

Supplementary Figure 2. Genetic associations with MAP-1 concentration in the *MASP1* gene region.

A-B. SNVs in the *MASP1* gene region were tested for their association with MAP-1 concentration in **(A**) matched controls (n=297) and (**B)** patients with SLE (n=384), separately. The locus zoom plots show unadjusted p values for individual SNVs. The dotted line indicates the significance threshold (adjusted $p<0.05$). r^2 indicates the Pearson correlation coefficient of allele count with the top SNV, indicated with a diamond shape.

C-D. Effects of top SNV rs80288719 on MAP-1 levels in serum. Data are presented as medians with IQR. Red horizontal lines in the graph indicate the median values.

C) Matched controls: MAP-1 levels were 371 (279-566) ng/mL for the T/T genotype (n=252), 277 (197-374) ng/mL for the T/C genotype (n=43), and 125 ng/mL for the C/C genotype (n=1), p value unadjusted 0.00034, adjusted 0.039.

D) SLE patients: MAP-1 levels were 435 (271-876) ng/mL for the T/T genotype (n=329), 298 (238-648) ng/mL for the T/C genotype (n=52), 109 ng/mL for the C/C genotype (n=3), p value unadjusted 0.0027, adjusted 0.21.

MAP, mannose-binding lectin-associated protein; MASP, mannose-binding lectin-associated serine protease; QTL, quantitative trait locus; SNV, single nucleotide variant.

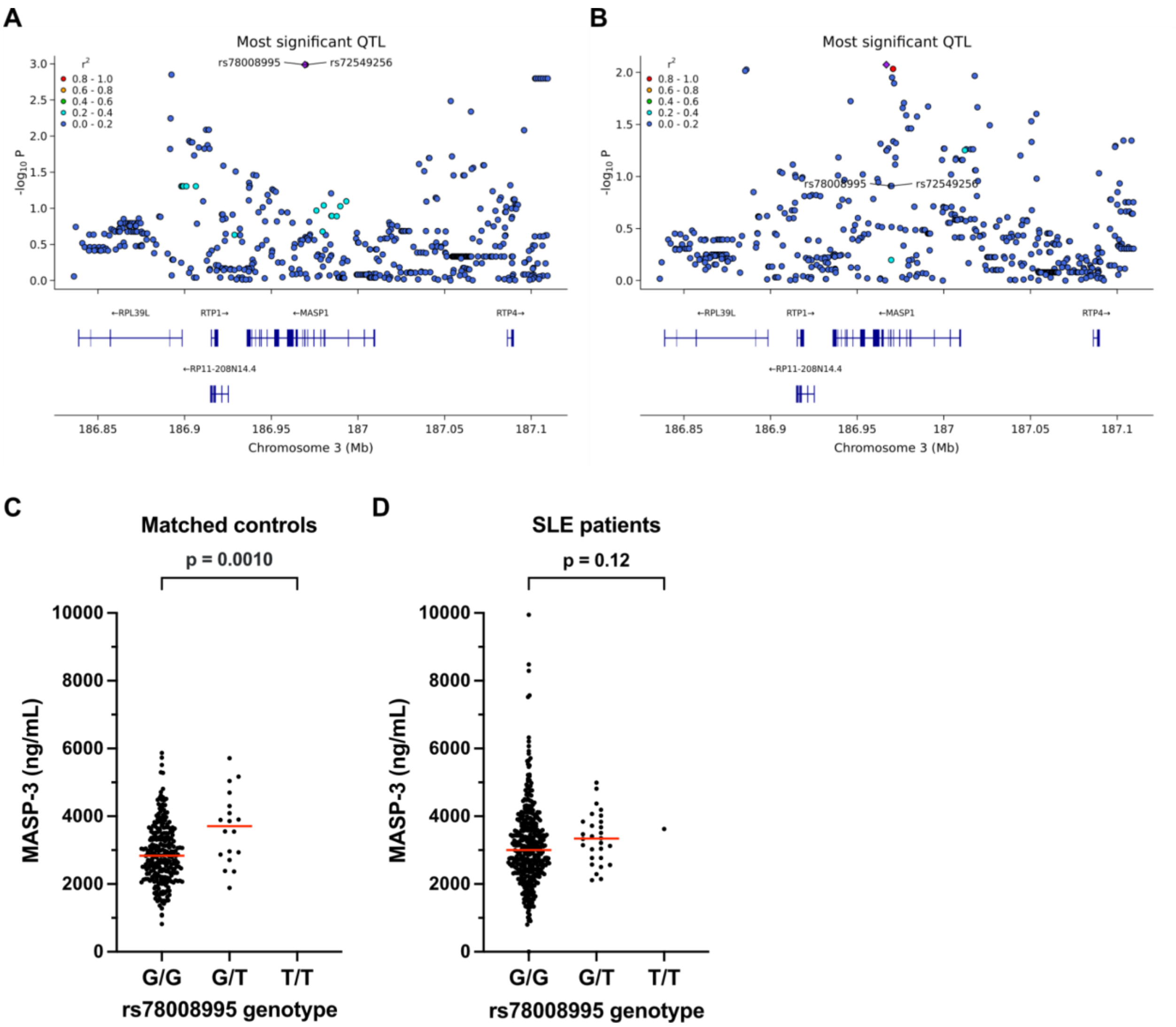

Supplementary Figure 3. Genetic associations with MASP-3 concentration in the *MASP1* gene region.

A-B. SNVs in the *MASP1* gene region were tested for their association with MASP-3 concentration in (**A**) matched controls (n=288) and (**B**) patients with SLE (n=421), separately. The locus zoom plots show unadjusted p values for individual SNVs. There were no SNVs above the significance threshold (adjusted $p<0.05$). r^2 indicates the Pearson correlation coefficient of allele count with the top SNV, indicated with a diamond shape.

C-D. Effect of the rs78008995 genotype on MASP-3 levels in serum. Data are presented as medians with IQR. Red horizontal lines in the graph indicate the median values.

C) Matched controls: MASP-3 levels were 2836 (2266-3394) ng/mL for the G/G genotype (n=266), and 3707 (2830-4399) ng/mL for the G/T genotype (n=18), p value unadjusted 0.0010, adjusted 0.085.

D) SLE patients: MASP-3 levels were 3003 (2404-3710) ng/mL for the G/G genotype (n=384), 3341 (2770-3839) ng/mL for the G/T genotype (n=28), and 3623 ng/mL for the T/T genotype (n=1), p value unadjusted 0.12, adjusted 1.0.
MASP, mannose-binding lectin-associated serine protease; QTL, quantitative trait locus; SNV, single nucleotide variant.

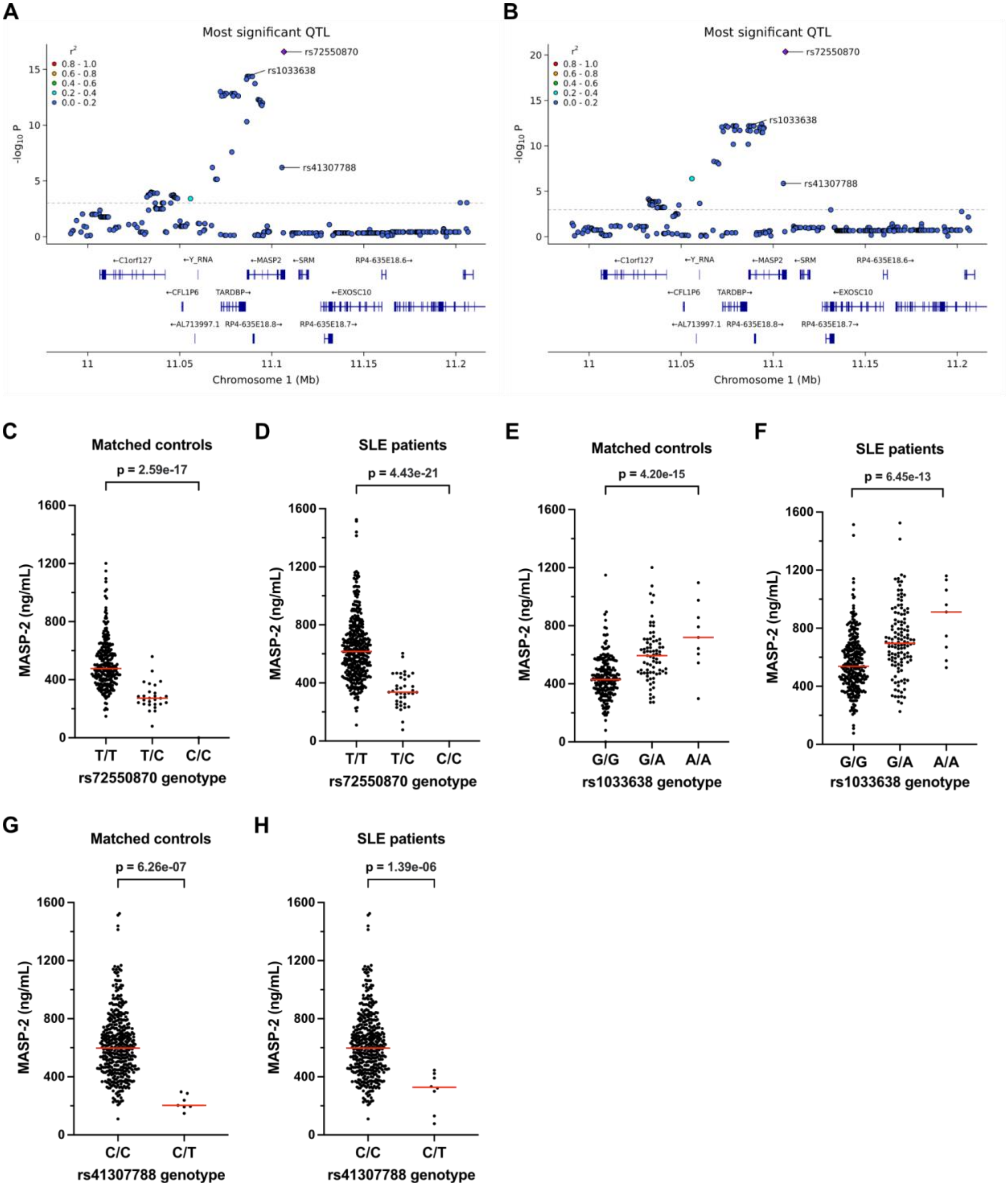

Supplementary Figure 4. Genetic associations with MASP-2 concentration in the *MASP2* gene region stratified by disease status.

A-B. SNVs in the *MASP2* gene region were tested for their association with MASP-2 concentration in **(A**) matched controls (n = 303) and (**B)** patients with SLE (n = 432), separately. The locus zoom plots show unadjusted p values for individual SNVs. The dotted line indicates

the significance threshold (adjusted p<0.05). r^2 indicates the Pearson correlation coefficient of allele count with the top SNV, indicated with a diamond shape.

C-D. Effects of the first independent pQTL rs72550870 on MASP-2 levels in serum.

C) Matched controls: MASP-2 levels were 477 (400-593) ng/mL for the T/T genotype (n=275), 272 (232-317) ng/mL for the T/C genotype (n=27), and 0 ng/mL for the C/C genotype (n=1), p value unadjusted 2.59e-17, adjusted <1e-04.

D) SLE patients: MASP-2 levels were 617 (479-749) ng/mL for the T/T genotype (n=394), and 336 (265-439) ng/mL for the T/C genotype (n=38), p value unadjusted 4.43e-21, adjusted <1e-04.

E-F. Effects of the second independent pQTL rs1033638 on MASP-2 levels in serum.

E) Matched controls: MASP-2 levels were 429 (355-515) ng/mL for the G/G genotype (n=218), 595 (475-693) ng/mL for the G/A genotype (n=75), and 719 (575-916) ng/mL for the A/A genotype (n=9), p value unadjusted 4.20e-15, adjusted <1e-04.

F) SLE patients: MASP-2 levels were 537 (431-664) ng/mL for the G/G genotype (n=297), 697 (575-848) ng/mL for the G/A genotype (n=126), and 911 (623-1098) ng/mL for the A/A genotype (n=9), p value unadjusted 6.45e-13, adjusted 0.0012.

G-H. Effects of the third independent pQTL rs41307788 on MASP-2 levels in serum.

G) Matched controls: MASP-2 levels were 467 (374-580) ng/mL for the C/C genotype (n=296), and 203 (192-286) ng/mL for the C/T genotype (n=7), p value unadjusted 6.26e-07, adjusted 0.0098.

H) SLE patients: MASP-2 levels were 597 (457-737) ng/mL for the C/C genotype (n = 424), and 328 (173-415) ng/mL for the C/T genotype (n=8), p value unadjusted 1.39e-06, adjusted 0.0014.

Data are presented as medians with IQR. Red horizontal lines in the graph indicate the median values. MASP, mannose-binding lectin-associated serine protease; QTL, quantitative trait locus; SNV, single nucleotide variant.

Supplementary Table 1. Serum levels of MASP-3 in patients with and without SLE disease manifestations and autoantibody specificities. P values <0.05, unadjusted for multiple comparisons, are shown in bold red.

	Patients without manifestation		Patients with manifestation		Mann Whitney p-value
	N	MASP-3 conc. Median (IQR), ng/mL	N	MASP-3 conc. Median (IQR), ng/mL	
**ACR 1982 Classification criteria (ever)**					
1 Malar rash	258	3124 (2484-3878)	243	2888 (2350-3502)	**0.0056**
2 Discoid rash	414	2999 (2423-3697)	87	3022 (2316-3836)	0.62
3 Photosensitivity	192	3006 (2325-3633)	309	2995 (2434-3733)	0.56
4 Oral ulcer	330	3034 (2430-3701)	171	2897 (2365-3746)	0.42
5 Arthritis	98	3088 (2431-4073)	403	2982 (2386-3623)	0.16
6 Serositis	302	3106 (2474-3807)	199	2865 (2308-3615)	**0.043**
6a Pleuritis	322	3082 (2464-3776)	179	2816 (2324-3613)	0.073
6b Pericarditis	409	3041 (2436-3803)	91	2755 (2208-3555)	**0.025**
7 Nephritis	305	3062 (2469-3785)	196	2884 (2336-3576)	**0.046**
8 Neurological disorder	447	3001 (2417-3717)	54	3037 (2401-3620)	0.87
8a Seizures	451	3001 (2417-3746)	50	3037 (2401-3569)	0.96
8b Psychosis	493	3006 (2420-3699)	8	2901 (2354-4559)	0.92
9 Haematological disorder	139	3108 (2429-3615)	362	2959 (2355-3776)	0.57
9a Haemolytic anaemia	471	3006 (2417-3700)	29	2777 (2365-3795)	0.99
9b Leucopenia	264	3102 (2423-3739)	237	2904 (2407-3697)	0.41
9c Lymphopenia	238	3054 (2423-3874)	263	2950 (2369-3613)	0.36
9d Thrombocytopenia	405	3038 (2423-3857)	96	2860 (2308-3406)	0.058
10 Immunological disorder	135	2969 (2432-3815)	367	3025 (2401-3670)	0.87
10b Anti-DNA	167	3086 (2503-3836)	334	2979 (2355-3643)	0.15
10c Anti-Sm	364	2994 (2418-3666)	134	3101 (2419-3901)	0.45
11 ANA	6	2957 (2047-3627)	496	3003 (2418-3710)	0.65
**Autoantibody positivity**					
dsDNA	316	3018 (2433-3739)	188	2954 (2351-3593)	0.37
Nucleosome	281	2945 (2353-3731)	223	3078 (2471-3676)	0.30
Ribosomal P	470	3006 (2410-3724)	34	2896 (2349-3523)	0.37
Sm	408	2994 (2371-3688)	96	3101 (2504-3889)	0.33
Sm RNP	367	3001 (2378-3717)	135	3006 (2436-3623)	0.92
RNP A	359	3001 (2369-3692)	128	3078 (2554-3839)	0.23
RNP 68	452	2994 (2366-3696)	52	3039 (2610-3880)	0.12
SSA Ro52	369	2871 (2328-3593)	135	3198 (2659-3907)	**0.00068**
SSA Ro60	302	2901 (2362-3703)	201	3070 (2431-3723)	0.23
SSB	395	2969 (2365-3670)	109	3062 (2487-3947)	0.17
β2GP1 IgG	374	3006 (2423-3801)	128	2911 (2332-3616)	0.36
β2GP1 IgM	471	2998 (2413-3700)	33	3153 (2231-3720)	0.95
β2GP1 IgA	419	2993 (2386-3774)	84	3069 (2467-3686)	0.66
ACL IgG	384	2999 (2390-3780)	118	3004 (2407-3665)	0.93
ACL IgM	478	2999 (2410-3703)	25	2995 (2073-3605)	0.73
ACL IgA	420	3006 (2414-3787)	83	2895 (2365-3594)	0.77
**IFN activity**					
High type I IFN activity	319	3001 (2423-3697)	131	3150 (2573-3931)	0.084
**Treatment**					
Corticosteroids	193	3038 (2445-3897)	310	2998 (2362-3594)	0.17
Antimalarials	286	3038 (2428-3698)	216	2972 (2357-3765)	0.54
DMARDs	296	3030 (2414-3778)	206	2988 (2375-3627)	0.55
Biologics	446	2999 (2410-3714)	51	3105 (2477-3640)	0.70

ACL: Anti-cardiolipin, ACR: American College of Rheumatology, ANA: Antinuclear antibody, dsDNA: double-stranded DNA, DMARDs: Disease-modifying anti-rheumatic drugs, IFN: interferon; MASP: Mannose-binding lectin-associated serine protease, RNP: Ribonucleoprotein, Sm: Smith antigen, SSA/B: Sjögren’s syndrome antigen A/B.

Supplementary Table 2. Serum levels of MAP-1 in patients with and without SLE disease manifestations and autoantibody specificities. P values <0.05, unadjusted for multiple comparisons, are shown in bold red.

	Patients without manifestation		Patients with manifestation		Mann Whitney p-value
	N	MAP-1 conc. Median (IQR), ng/mL	N	MAP-1 conc. Median (IQR), ng/mL	
**ACR 1982 Classification criteria (ever)**					
1 Malar rash	249	455 (261-953)	212	432 (266-761)	0.49
2 Discoid rash	386	428 (258-796)	75	561 (304-1155)	**0.0045**
3 Photosensitivity	178	471 (272-841)	283	426 (261-884)	0.41
4 Oral ulcer	306	430 (259-805)	155	481 (270-960)	0.24
5 Arthritis	94	495 (272-847)	367	437 (261-884)	0.61
6 Serositis	283	470 (288-903)	178	416 (242-819)	0.070
6a Pleuritis	301	470 (285-898)	160	394 (234-819)	**0.044**
6b Pericarditis	382	452 (265-847)	78	416 (253-915)	0.51
7 Nephritis	280	448 (273-880)	181	441 (237-810)	0.45
8 Neurological disorder	412	450 (264-863)	49	395 (250-759)	0.45
8a Seizures	415	450 (266-848)	46	386 (241-855)	0.37
8b Psychosis	454	441 (261-872)	7	459 (329-629)	0.98
9 Haematological disorder	121	405 (210-739)	340	468 (289-937)	**0.013**
9a Haemolytic anaemia	433	447 (265-844)	27	387 (226-946)	0.99
9b Leucopenia	240	408 (226-812)	221	481 (295-987)	**0.0094**
9c Lymphopenia	216	401 (234-740)	245	498 (289-987)	**0.0036**
9d Thrombocytopenia	371	450 (261-848)	90	416 (268-842)	0.57
10 Immunological disorder	128	375 (232-674)	334	481 (289-948)	**0.0054**
10b Anti-DNA	157	440 (259-865)	304	448 (271-846)	0.53
10c Anti-Sm	341	397 (246-675)	117	643 (376-1361)	**<0.0001**
11 ANA	6	457 (130-877)	456	442 (264-848)	0.59
**Autoantibody positivity**					
dsDNA	290	432 (260-762)	174	517 (275-986)	**0.041**
Nucleosome	258	399 (229-672)	206	527 (303-1043)	**<0.0001**
Ribosomal P	434	430 (261-816)	30	769 (425-1271)	**0.0026**
Sm	380	405 (251-703)	84	776 (472-1412)	**<0.0001**
Sm RNP	344	405 (247-678)	119	619 (375-1280)	**<0.0001**
RNP A	334	411 (252-760)	113	603 (360-1243)	**<0.0001**
RNP 68	417	420 (256-791)	47	816 (441-1447)	**<0.0001**
SSA Ro52	340	407 (248-786)	124	558 (350-1053)	**0.00012**
SSA Ro60	279	409 (243-720)	184	518 (306-1047)	**0.00062**
SSB	363	414 (254-763)	101	622 (371-1230)	**<0.0001**
β2GP1 IgG	342	468 (263-962)	120	389 (262-601)	**0.015**
β2GP1 IgM	432	442 (264-880)	32	426 (245-677)	0.50
β2GP1 IgA	385	458 (266-949)	78	391 (247-582)	**0.029**
ACL IgG	351	459 (261-960)	111	375 (266-609)	**0.029**
ACL IgM	437	441 (265-886)	26	426 (222-633)	0.34
ACL IgA	387	453 (270-946)	76	355 (212-606)	**0.013**
**IFN activity**					
High type I IFN activity	297	367 (231-563)	115	801 (455-1574)	**<0.0001**
**Treatment**					
Corticosteroids	180	436 (261-804)	283	455 (267-910)	0.44
Antimalarials	262	445 (256-856)	200	445 (270-846)	0.97
DMARDs	275	458 (275-990)	188	413 (253-713)	0.068
Biologics	410	450 (263-899)	47	381 (261-597)	0.097

ACL: Anti-cardiolipin, ACR: American College of Rheumatology, ANA: Antinuclear antibody, dsDNA: double-stranded DNA, DMARDs: Disease-modifying anti-rheumatic drugs, IFN: interferon; MAP: Mannose-binding lectin-associated protein, RNP: Ribonucleoprotein, Sm: Smith antigen, SSA/B: Sjögren’s syndrome antigen A/B.

Supplementary Table 3. Serum levels of MASP-2 in patients with and without SLE disease manifestations and autoantibody specificities. P values <0.05, unadjusted for multiple comparisons, are shown in bold red.

	Patients without manifestation		Patients with manifestation		Mann Whitney p-value
	N	MASP-2 conc. Median (IQR), ng/mL	N	MASP-2 conc. Median (IQR), ng/mL	
**ACR 1982 Classification criteria (ever)**					
1 Malar rash	263	598 (451-728)	249	570 (447-723)	0.40
2 Discoid rash	424	580 (446-730)	88	605 (495-714)	0.15
3 Photosensitivity	196	588 (448-756)	316	587 (452-708)	0.81
4 Oral ulcer	339	593 (460-731)	173	563 (447-703)	0.31
5 Arthritis	99	599 (447-706)	413	587 (452-728)	0.70
6 Serositis	308	586 (448-722)	204	592 (459-736)	0.62
6a Pleuritis	328	578 (448-721)	184	606 (461-745)	0.35
6b Pericarditis	419	589 (451-728)	92	560 (439-704)	0.75
7 Nephritis	309	556 (445-721)	203	618 (475-739)	0.058
8 Neurological disorder	457	580 (447-723)	55	633 (514-737)	**0.047**
8a Seizures	462	579 (447-723)	50	639 (528-740)	**0.024**
8b Psychosis	503	589 (450-727)	9	514 (445-778)	0.66
9 Haematological disorder	142	579 (454-734)	370	590 (450-723)	0.76
9a Haemolytic anaemia	481	587 (449-732)	29	606 (473-678)	0.80
9b Leucopenia	267	577 (446-705)	245	602 (465-733)	0.41
9c Lymphopenia	241	581 (448-722)	271	591 (453-732)	0.75
9d Thrombocytopenia	413	589 (455-729)	99	587 (437-721)	0.70
10 Immunological disorder	137	566 (449-715)	376	597 (457-728)	0.64
10b Anti-DNA	170	567 (448-722)	342	603 (463-732)	0.45
10c Anti-Sm	372	586 (459-727)	137	613 (448-729)	0.46
11 ANA	6	600 (457-657)	507	587 (450-727)	0.82
**Autoantibody positivity**					
dsDNA	322	577 (448-722)	193	614 (464-736)	0.40
Nucleosome	287	577 (450-704)	228	604 (454-742)	0.40
Ribosomal P	480	587 (452-731)	35	615 (445-670)	0.67
Sm	418	582 (455-723)	97	622 (442-749)	0.35
Sm RNP	377	568 (447-708)	136	627 (490-748)	**0.030**
RNP A	369	586 (450-732)	128	620 (482-726)	0.23
RNP 68	463	585 (448-724)	52	623 (486-759)	0.19
SSA Ro52	375	567 (447-691)	140	646 (465-795)	**0.0046**
SSA Ro60	306	567 (443-694)	208	621 (466-759)	**0.031**
SSB	401	568 (447-697)	114	650 (467-795)	**0.0048**
β2GP1 IgG	383	602 (456-741)	130	551 (448-677)	**0.028**
β2GP1 IgM	481	589 (452-731)	34	534 (448-677)	0.31
β2GP1 IgA	429	597 (456-737)	85	543 (433-674)	0.052
ACL IgG	394	602 (459-742)	119	543 (445-672)	**0.016**
ACL IgM	488	589 (452-730)	26	534 (448-672)	0.39
ACL IgA	430	596 (456-737)	84	542 (431-676)	0.058
**IFN activity**					
High type I IFN activity	325	569 (450-701)	134	660 (514-795)	**0.00018**
**Treatment**					
Corticosteroids	196	558 (449-681)	318	604 (455-739)	0.17
Antimalarials	295	614 (490-749)	218	532 (422-696)	**0.00020**
DMARDs	300	591 (456-722)	212	585 (437-732)	0.75
Biologics	456	585 (450-726)	52	601 (434-726)	0.92

ACL: Anti-cardiolipin, ACR: American College of Rheumatology, ANA: Antinuclear antibody, dsDNA: double-stranded DNA, DMARDs: Disease-modifying anti-rheumatic drugs, IFN: interferon, MASP: Mannose-binding lectin-associated serine protease, RNP: Ribonucleoprotein, Sm: Smith antigen, SSA/B: Sjögren’s syndrome antigen A/B.

Supplementary Table 4. Associations between MASP-3 and clinical manifestations.
MASP-3 concentration was categorized into quartiles, and logistic regression models were used to calculate odds ratios (ORs) for disease manifestations and autoantibodies in the highest quartile (Q4) compared to the lowest quartile (Q1). Analyses were adjusted for sex, age at follow-up, and high type I interferon activity. P values <0.05, unadjusted for multiple comparisons, are shown in bold red.

	Total N	Q1 n (%)	Q2 n (%)	Q3 n (%)	Q4 n (%)	Adjusted for age and sex		Adjusted for age, sex and high IFN score	
						OR (95% CI)	p-value	OR (95% CI)	p-value
**ACR 1982 classification criteria (ever)**									
1 Malar rash	501	68 (55)	68 (54)	58 (46)	49 (39)	0.49 (0.30-0.82)	**0.0069**	0.53 (0.31-0.91)	**0.022**
2 Discoid rash	501	23 (19)	20 (16)	20 (16)	24 (19)	1.1 (0.57-2.1)	0.80	1.0 (0.52-2.0)	0.94
3 Photosensitivity	501	70 (56)	86 (68)	74 (59)	79 (63)	1.3 (0.79-2.3)	0.29	1.2 (0.67-2.1)	0.55
4 Oral ulcer	501	48 (39)	44 (35)	36 (29)	43 (34)	0.77 (0.46-1.3)	0.33	0.68 (0.39-1.2)	0.18
5 Arthritis	501	102 (82)	103 (81)	105 (84)	93 (74)	0.58 (0.31-1.1)	0.082	0.48 (0.24-0.95)	**0.034**
6 Serositis	501	60 (48)	47 (37)	48 (38)	44 (35)	0.61 (0.36-1.0)	0.062	0.57 (0.33-0.99)	**0.047**
6a Pleuritis	501	54 (44)	44 (35)	40 (32)	41 (33)	0.67 (0.40-1.1)	0.13	0.60 (0.34-1.0)	0.068
6b Pericarditis	500	31 (25)	22 (17)	21 (17)	17 (14)	0.48 (0.25-0.94)	**0.032**	0.48 (0.24-0.98)	**0.042**
7 Nephritis	501	57 (46)	50 (39)	45 (36)	44 (35)	0.61 (0.36-1.0)	0.066	0.58 (0.33-1.0)	0.053
8 Neurological disorder	501	13 (10)	14 (11)	15 (12)	12 (9.6)	0.93 (0.40-2.1)	0.86	0.85 (0.35-2.0)	0.71
8a Seizures	501	12 (9.7)	13 (10)	15 (12)	10 (8.0)	0.81 (0.34-2.0)	0.65	0.73 (0.29-1.9)	0.51
8b Psychosis	501	3 (2.4)	2 (1.6)	0 (0.0)	3 (2.4)	1.1 (0.21-5.5)	0.95	0.99 (0.19-5.2)	0.99
9 Haematological disorder	501	93 (75)	96 (76)	79 (63)	94 (75)	0.92 (0.51-1.7)	0.78	0.89 (0.47-1.7)	0.72
9a Haemolytic anaemia	500	8 (6.5)	7 (5.6)	6 (4.8)	8 (6.4)	0.90 (0.32-2.5)	0.84	1.3 (0.42-4.2)	0.63
9b Leucopenia	501	59 (48)	69 (54)	51 (41)	58 (46)	0.92 (0.56-1.5)	0.75	0.97 (0.56-1.7)	0.90
9c Lymphopenia	501	67 (54)	71 (56)	65 (52)	60 (48)	0.72 (0.43-1.2)	0.21	0.65 (0.38-1.1)	0.12
9d Thrombocytopenia	501	26 (21)	30 (24)	29 (23)	11 (8.8)	0.37 (0.18-0.80)	**0.011**	0.37 (0.17-0.80)	**0.012**
10 Immunological disorder	502	92 (74)	89 (70)	99 (79)	87 (70)	0.68 (0.38-1.2)	0.20	0.59 (0.32-1.1)	0.099
10b Anti-DNA	501	90 (73)	81 (64)	86 (68)	77 (62)	0.53 (0.30-0.92)	**0.024**	0.48 (0.27-0.87)	**0.016**
10c Anti-Sm	498	33 (27)	31 (24)	31 (25)	39 (31)	1.0 (0.59-1.9)	0.88	0.91 (0.47-1.8)	0.79
11 ANA	502	122 (98)	126 (99)	124 (98)	124 (99)	1.9 (0.16-21)	0.62	2.0 (0.17-22)	0.59
**Autoantibody positivity**									
dsDNA	504	53 (42)	45 (35)	46 (37)	44 (35)	0.62 (0.37-1.1)	0.083	0.51 (0.28-0.92)	**0.025**
Nucleosome	504	50 (40)	56 (44)	63 (50)	54 (43)	1.0 (0.58-1.7)	0.96	0.65 (0.36-1.2)	0.16
Ribosomal P	504	9 (7.1)	10 (7.9)	9 (7.1)	6 (4.8)	0.57 (0.19-1.7)	0.31	0.44 (0.14-1.3)	0.14

	Total N	Q1 n (%)	Q2 n (%)	Q3 n (%)	Q4 n (%)	Adjusted for age and sex		Adjusted for age, sex and high IFN score	
						**OR (95% CI)**	**p-value**	**OR (95% CI)**	**p-value**
Sm	504	21 (17)	25 (20)	23 (18)	27 (22)	1.1 (0.57-2.1)	0.77	0.89 (0.42–1.9)	0.77
Sm RNP	502	30 (24)	37 (29)	35 (28)	33 (26)	1.0 (0.56-1.8)	1.00	0.70 (0.37-1.3)	0.28
RNP A	487	24 (21)	35 (29)	32 (26)	37 (30)	1.5 (0.84-2.8)	0.16	1.2 (0.64-2.3)	0.56
RNP 68	504	8 (6.3)	16 (13)	13 (10)	15 (12)	1.7 (0.67-4.2)	0.27	1.2 (0.46-3.2)	0.68
SSA Ro52	504	22 (17)	30 (24)	42 (33)	41 (33)	2.5 (1.4-4.5)	**0.0031**	2.0 (1.1- 3.8)	**0.030**
SSA Ro60	503	46 (37)	48 (38)	57 (45)	50 (40)	1.2 (0.71-2.0)	0.53	1.1 (0.64-2.0)	0.68
SSB	504	22 (17)	29 (23)	27 (21)	31 (25)	1.6 (0.84-2.9)	0.15	1.6 (0.81-3.1)	0.18
β2GP1 IgG	502	35 (28)	31 (25)	34 (27)	28 (22)	0.75 (0.42-1.3)	0.32	0.80 (0.43-1.5)	0.48
β2GP1 IgM	504	9 (7.1)	7 (5.5)	9 (7.1)	8 (6.4)	0.89 (0.33-2.4)	0.82	0.93 (0.33-2.6)	0.89
β2GP1 IgA	503	20 (16)	21 (17)	23 (18)	20 (16)	1.1 (0.53-2.1)	0.89	1.3 (0.61-2.6)	0.52
ACL IgG	502	29 (23)	30 (24)	31 (25)	28 (22)	1.0 (0.53-1.7)	0.90	0.93 (0.49-1.8)	0.83
ACL IgM	503	7 (5.6)	6 (4.7)	7 (5.6)	5 (4.0)	0.71 (0.22-2.3)	0.57	0.78 (0.23-2.6)	0.68
ACL IgA	503	22 (17)	22 (17)	22 (18)	17 (14)	0.77 (0.39-1.5)	0.46	0.91 (0.44-1.9)	0.80

ACL: Anti-cardiolipin, ACR: American College of Rheumatology, ANA: Antinuclear antibody, dsDNA: double-stranded DNA, IFN: interferon, MASP: Mannose-binding lectin-associated serine protease, RNP: Ribonucleoprotein, Sm: Smith antigen, SSA/B: Sjögren’s syndrome antigen A/B.

Supplementary Table 5. Associations between MAP-1 and clinical manifestations.
MAP-1 concentration was categorized into quartiles, and logistic regression models were used to calculate odds ratios (ORs) for disease manifestations and autoantibodies in the highest quartile (Q4) compared to the lowest quartile (Q1). Analyses were adjusted for sex, age at follow-up, and high type I interferon activity. P values <0.05, unadjusted for multiple comparisons, are shown in bold red.

	Total N	Q1 n (%)	Q2 n (%)	Q3 n (%)	Q4 n (%)	Adjusted for age and sex		Adjusted for age, sex and high IFN score	
						OR (95% CI)	p-value	OR (95% CI)	p-value
**ACR 1982 classification criteria (ever)**									
1 Malar rash	461	52 (45)	59 (52)	53 (46)	48 (41)	0.81 (0.48-1.4)	0.44	0.82 (0.46-1.5)	0.50
2 Discoid rash	461	14 (12)	15 (13)	17 (15)	29 (25)	2.8 (1.4-5.7)	**0.0050**	2.7 (1.2-5.8)	**0.012**
3 Photosensitivity	461	73 (63)	76 (67)	61 (53)	73 (63)	1.0 (0.57-1.7)	0.99	1.0 (0.56-1.9)	0.91
4 Oral ulcer	461	35 (30)	36 (32)	39 (34)	45 (39)	1.4 (0.78-2.3)	0.28	1.1 (0.58-2.0)	0.81
5 Arthritis	461	94 (81)	93 (82)	88 (77)	92 (79)	0.83 (0.43-1.6)	0.58	0.60 (0.29-1.2)	0.16
6 Serositis	461	54 (47)	41 (36)	41 (36)	42 (36)	0.71 (0.42-1.2)	0.22	0.59 (0.32-1.1)	0.086
6a Pleuritis	461	51 (44)	36 (32)	35 (30)	38 (33)	0.69 (0.40-1.2)	0.17	0.52 (0.28-0.96)	**0.035**
6b Pericarditis	460	20 (17)	22 (19)	16 (14)	20 (17)	1.1 (0.53-2.2)	0.84	1.0 (0.45-2.2)	1.00
7 Nephritis	461	51 (44)	40 (35)	47 (41)	43 (37)	0.72 (0.42-1.2)	0.24	0.52 (0.28-0.95)	**0.034**
8 Neurological disorder	461	13 (11)	14 (12)	11 (10)	11 (9.5)	0.86 (0.37-2.0)	0.73	1.1 (0.42-2.7)	0.89
8a Seizures	461	13 (11)	14 (12)	8 (7.0)	11 (9.5)	0.84 (0.35-2.0)	0.68	1.0 (0.39-2.6)	1.00
8b Psychosis	461	1 (0.9)	1 (0.9)	5 (4.3)	0 (0.0)	n/a		n/a	
9 Haematological disorder	461	71 (61)	89 (78)	89 (77)	91 (78)	2.1 (1.1-3.7)	**0.016**	1.5 (0.76-2.8)	0.26
9a Haemolytic anaemia	460	7 (6.1)	7 (6.1)	5 (4.3)	8 (6.9)	1.0 (0.35-2.9)	0.98	0.99 (0.31-3.1)	0.98
9b Leucopenia	461	42 (36)	57 (50)	59 (51)	63 (54)	2.0 (1.2-3.5)	**0.0089**	1.4 (0.79-2.6)	0.24
9c Lymphopenia	461	49 (42)	55 (48)	71 (62)	70 (60)	1.9 (1.1-3.3)	**0.015**	1.5 (0.83-2.7)	0.18
9d Thrombocytopenia	461	21 (18)	26 (23)	21 (18)	22 (19)	1.1 (0.57-2.2)	0.77	1.2 (0.56-2.4)	0.69
10 Immunological disorder	462	73 (63)	82 (71)	89 (77)	90 (78)	1.7 (0.93-3.1)	0.088	0.96 (0.50-1.9)	0.92
10b Anti-DNA	461	72 (63)	79 (69)	77 (67)	76 (66)	1.0 (0.55-1.7)	0.88	0.64 (0.34-1.2)	0.16
10c Anti-Sm	458	18 (16)	18 (16)	37 (32)	44 (39)	2.9 (1.5-5.5)	**0.0015**	1.3 (0.64-2.7)	0.46
11 ANA	462	114 (98)	113 (99)	114 (98)	115 (99)	1.7 (0.15-19)	0.67	0.99 (0.087-11)	0.99
**Autoantibody positivity**									
dsDNA	464	39 (34)	42 (37)	40 (34)	53 (46)	1.4 (0.79-2.4)	0.26	0.99 (0.53-1.9)	0.98
Nucleosome	464	36 (31)	51 (44)	52 (44)	67 (58)	2.6 (1.5-4.6)	**0.00063**	1.7 (0.90-3.1)	0.11
Ribosomal P	464	4 (3.4)	4 (3.5)	8 (6.8)	14 (12)	3.3 (1.0-10)	**0.044**	3.5 (0.90-14)	0.071

	Total N	Q1 n (%)	Q2 n (%)	Q3 n (%)	Q4 n (%)	Adjusted for age and sex		Adjusted for age, sex and high IFN score	
						**OR (95% CI)**	**p-value**	**OR (95% CI)**	**p-value**
Sm	464	11 (9.5)	9 (7.8)	28 (24)	36 (31)	3.6 (1.7-7.7)	**0.00094**	1.7 (0.71-3.9)	0.24
Sm RNP	463	20 (17)	20 (18)	32 (27)	47 (41)	2.9 (1.6-5.4)	**0.00066**	1.6 (0.80-3.2)	0.18
RNP A	447	19 (17)	23 (21)	31 (28)	40 (35)	2.4 (1.3-4.6)	**0.0060**	1.5 (0.76-3.0)	0.24
RNP 68	464	4 (3.4)	8 (7.0)	12 (10)	23 (20)	6.1 (2.0-18)	**0.0015**	3.3 (1.0-11)	**0.049**
SSA Ro52	464	19 (16)	24 (21)	40 (34)	41 (35)	3.1 (1.6-5.8)	**0.00051**	2.9 (1.4-5.8)	**0.0031**
SSA Ro60	463	33 (29)	44 (38)	50 (43)	57 (49)	2.6 (1.5-4.5)	**0.00084**	2.1 (1.1-3.9)	**0.018**
SSB	464	17 (15)	18 (16)	27 (23)	39 (34)	3.0 (1.6-5.8)	**0.00082**	3.2 (1.6-6.6)	**0.0014**
β2GP1 IgG	462	30 (26)	39 (34)	34 (29)	17 (15)	0.50 (0.26-1.0)	**0.040**	0.52 (0.25-1.1)	0.087
β2GP1 IgM	464	8 (6.9)	8 (7.0)	11 (9.4)	5 (4.3)	0.65 (0.20-2.1)	0.47	0.31 (0.17-2.2)	0.45
β2GP1 IgA	463	21 (18)	25 (22)	24 (21)	8 (6.9)	0.34 (0.14-0.81)	**0.015**	0.32 (0.12-0.85)	**0.022**
ACL IgG	462	27 (23)	38 (33)	30 (26)	16 (14)	0.52 (0.26-1.0)	0.065	0.51 (0.24-1.1)	0.089
ACL IgM	463	7 (6.0)	6 (5.2)	10 (8.6)	3 (2.6)	0.43 (0.11-1.7)	0.24	0.33 (0.063-1.7)	0.18
ACL IgA	463	24 (21)	20 (17)	24 (21)	8 (6.9)	0.29 (0.12-0.68)	**0.0044**	0.29 (0.11-0.76)	**0.011**

ACL: Anti-cardiolipin, ACR: American College of Rheumatology, ANA: Antinuclear antibody, dsDNA: double-stranded DNA, IFN: interferon, MAP: Mannose-binding lectin-associated protein, RNP: Ribonucleoprotein, Sm: Smith antigen, SSA/B: Sjögren's syndrome antigen A/B.

Supplementary Table 6. Associations between MASP-2 and clinical manifestations.
MASP-2 concentration was categorized into quartiles, and logistic regression models were used to calculate odds ratios (ORs) for disease manifestations and autoantibodies in the highest quartile (Q4) compared to the lowest quartile (Q1). Analyses were adjusted for sex,age at follow-up, and high type I interferon activity. P values <0.05, unadjusted for multiple comparisons, are shown in bold red.

	Total	Q1	Q2	Q3	Q4	Adjusted for age and sex		Adjusted for age, sex and high IFN score	
	N	n (%)	n (%)	n (%)	n (%)	OR (95% CI)	p-value	OR (95% CI)	p-value
**ACR 1982 classification criteria (ever)**									
1 Malar rash	512	63 (49)	68 (53)	58 (45)	60 (47)	0.99 (0.60-1.6)	0.97	1.0 (0.58-1.7)	1.00
2 Discoid rash	512	14 (11)	25 (20)	30 (23)	19 (15)	1.4 (0.69-3.1)	0.33	0.93 (0.42-2.0)	0.85
3 Photosensitivity	512	79 (61)	80 (63)	85 (66)	72 (57)	0.86 (0.51-1.4)	0.57	0.91 (0.52-1.6)	0.74
4 Oral ulcer	512	47 (36)	46 (36)	40 (31)	40 (31)	0.85 (0.50-1.4)	0.54	0.84 (0.48-1.5)	0.55
5 Arthritis	512	103 (80)	105 (82)	101 (79)	104 (82)	1.2 (0.65-2.3)	0.52	1.0 (0.50-2.0)	1.00
6 Serositis	512	48 (37)	52 (41)	50 (39)	54 (43)	1.2 (0.71-2.0)	0.53	1.0 (0.57-1.7)	0.93
6a Pleuritis	512	42 (33)	44 (34)	48 (38)	50 (39)	1.3 (0.76-2.1)	0.36	0.98 (0.56-1.7)	0.93
6b Pericarditis	511	24 (19)	24 (19)	23 (18)	21 (17)	0.81 (0.42-1.6)	0.54	0.84 (0.41-1.7)	0.63
7 Nephritis	512	44 (34)	46 (36)	59 (46)	54 (43)	1.4 (0.84-2.4)	0.19	1.2 (0.70-2.1)	0.48
8 Neurological disorder	512	8 (6.2)	14 (11)	17 (13)	16 (13)	2.2 (0.89-5.3)	0.088	2.9 (1.1-7.8)	**0.036**
8a Seizures	512	6 (4.7)	13 (10)	16 (13)	15 (12)	2.8 (1.0-7.4)	**0.042**	4.3 (1.3-13)	**0.014**
8b Psychosis	512	2 (1.6)	4 (3.1)	1 (0.8)	2 (1.6)	1.0 (0.14-7.4)	1.0	0.81 (0.10-6.3)	0.84
9 Haematological disorder	512	94 (73)	90 (70)	96 (75)	90 (71)	0.98 (0.56-1.7)	0.94	0.94 (0.51-1.7)	0.85
9a Haemolytic anaemia	510	6 (4.7)	7 (5.6)	12 (9.4)	4 (3.1)	0.7 (0.20-2.6)	0.62	0.53 (0.12-2.3)	0.39
9b Leucopenia	512	58 (45)	59 (46)	65 (51)	63 (50)	1.2 (0.76-2.0)	0.38	1.3 (0.78-2.3)	0.29
9c Lymphopenia	512	67 (52)	67 (52)	68 (53)	69 (54)	1.2 (0.73-2.0)	0.47	1.0 (0.58-1.7)	1.00
9d Thrombocytopenia	512	27 (21)	24 (19)	25 (20)	23 (18)	0.81 (0.43-1.5)	0.50	0.59 (0.30-1.2)	0.13
10 Immunological disorder	513	92 (72)	90 (70)	100 (78)	94 (74)	1.2 (0.69-2.2)	0.50	1.0 (0.54-1.9)	0.97
10b Anti-DNA	512	81 (64)	81 (63)	92 (71)	88 (69)	1.4 (0.81-2.4)	0.24	1.2 (0.67-2.2)	0.54
10c Anti-Sm	509	35 (28)	29 (22)	39 (30)	34 (27)	1.1 (0.59-1.9)	0.84	0.69 (0.35-1.4)	0.28
11 ANA	513	128 (99)	127 (99)	126 (98)	126 (99)	1.0 (0.06-17.1)	0.97	0.93 (0.056-15)	0.96
**Autoantibody positivity**									
dsDNA	515	45 (35)	44 (34)	53 (41)	51 (40)	1.4 (0.82-2.4)	0.22	1.2 (0.67-2.2)	0.53
Nucleosome	515	56 (43)	52 (40)	57 (44)	63 (49)	1.5 (0.87-2.4)	0.16	1.0 (0.57-1.9)	0.92
Ribosomal P	515	9 (7.0)	7 (5.4)	14 (11)	5 (3.9)	0.59 (0.19-1.8)	0.35	0.51 (0.15-1.7)	0.28

	Total N	Q1 n (%)	Q2 n (%)	Q3 n (%)	Q4 n (%)	Adjusted for age and sex		Adjusted for age, sex and high IFN score	
						OR (95% CI)	p-value	OR (95% CI)	p-value
Sm	515	27 (21)	16 (12)	29 (22)	25 (20)	1.1 (0.56-2.0)	0.86	0.61 (0.29-1.3)	0.20
Sm RNP	513	27 (21)	31 (24)	41 (32)	37 (29)	1.6 (0.92-3.0)	0.093	1.1 (0.57-2.1)	0.76
RNP A	497	25 (21)	31 (25)	41 (32)	31 (25)	1.3 (0.70-2.4)	0.41	0.96 (0.49-1.9)	0.91
RNP 68	515	9 (7.0)	13 (10)	16 (12)	14 (11)	1.8 (0.76-4.5)	0.18	1.2 (0.44-3.1)	0.74
SSA Ro52	515	31 (24)	23 (18)	36 (28)	50 (39)	2.0 (1.2-3.4)	**0.013**	1.5 (0.83-2.6)	0.18
SSA Ro60	514	44 (34)	48 (38)	53 (41)	63 (49)	1.9 (1.1-3.1)	**0.015**	1.6 (0.92-2.8)	0.094
SSB	515	23 (18)	18 (14)	33 (26)	40 (31)	2.1 (1.2-3.9)	**0.011**	2.0 (1.1-3.9)	**0.028**
β2GP1 IgG	513	36 (28)	38 (29)	33 (26)	23 (18)	0.56 (0.31-1.0)	0.054	0.64 (0.33-1.2)	0.18
β2GP1 IgM	515	9 (7.0)	9 (7.0)	11 (8.5)	5 (3.9)	0.54 (0.17-1.7)	0.28	0.45 (0.13-1.6)	0.21
β2GP1 IgA	514	25 (19)	25 (19)	22 (17)	13 (10)	0.45 (0.22-0.94)	**0.033**	0.42 (0.18-0.98)	**0.043**
ACL IgG	513	35 (27)	34 (26)	31 (24)	19 (15)	0.46 (0.25-0.87)	**0.016**	0.50 (0.25-0.99)	**0.047**
ACL IgM	514	7 (5.4)	7 (5.5)	8 (6.2)	4 (3.1)	0.56 (0.16-2.0)	0.36	0.47 (0.11-1.9)	0.30
ACL IgA	514	25 (19)	25 (19)	21 (16)	13 (10)	0.45 (0.22-0.94)	**0.033**	0.49 (0.22-1.1)	0.082

ACL: Anti-cardiolipin, ACR: American College of Rheumatology, ANA: Antinuclear antibody, dsDNA: double-stranded DNA, IFN: interferon, MASP: Mannose-binding lectin-associated serine protease, RNP: Ribonucleoprotein, Sm: Smith antigen, SSA/B: Sjögren's syndrome antigen A/B.

Supplementary Table 7. Associations between C1q and clinical manifestations.
C1q concentration was categorized into quartiles, and logistic regression models were used to calculate odds ratios (ORs) for disease manifestations and autoantibodies in the highest quartile (Q4) compared to the lowest quartile (Q1). Analyses were adjusted for sex and age at follow-up. P values <0.05, unadjusted for multiple comparisons, are shown in bold red.

	Total N	Q1 n (%)	Q2 n (%)	Q3 n (%)	Q4 n (%)	OR (95% CI)	p-value
**ACR 1982 classification criteria**							
1 Malar rash	477	46 (39)	67 (56)	61 (51)	58 (49)	1.6 (0.95-2.8)	0.078
2 Discoid rash	477	15 (13)	30 (25)	16 (13)	26 (22)	1.6 (0.77-3.2)	0.21
3 Photosensitivity	477	72 (61)	69 (58)	75 (63)	85 (71)	1.4 (0.81-2.6)	0.21
4 Oral ulcer	477	38 (32)	34 (28)	45 (38)	41 (34)	1.2 (0.71-2.2)	0.44
5 Arthritis	477	97 (82)	99 (83)	101 (85)	90 (76)	0.76 (0.40-1.4)	0.41
6 Serositis	477	42 (35)	45 (38)	52 (44)	51 (43)	1.3 (0.75-2.2)	0.35
6a Pleuritis	477	39 (33)	42 (35)	46 (39)	47 (39)	1.2 (0.71-2.1)	0.47
6b Pericarditis	476	18 (15)	19 (16)	27 (23)	19 (16)	1.1 (0.52-2.2)	0.86
7 Nephritis	477	55 (46)	49 (41)	44 (37)	42 (35)	0.70 (0.41-1.2)	0.20
8 Neurological disorder	477	13 (11)	9 (7.5)	14 (12)	17 (14)	1.3 (0.58-2.9)	0.53
8a Seizures	477	12 (10)	9 (7.5)	12 (10)	15 (13)	1.3 (0.57-3.0)	0.53
8b Psychosis	477	1 (0.8)	1 (0.8)	3 (2.5)	4 (3.4)	2.8 (0.30-26)	0.36
9 Haematological disorder	477	93 (78)	94 (78)	85 (71)	76 (64)	0.56 (0.31-1.0)	0.053
9a Haemolytic anaemia	475	9 (7.6)	6 (5.0)	9 (7.6)	5 (4.2)	0.62 (0.20-2.0)	0.42
9b Leucopenia	477	64 (54)	57 (48)	57 (48)	49 (41)	0.63 (0.37-1.1)	0.086
9c Lymphopenia	477	70 (59)	65 (54)	65 (55)	55 (46)	0.63 (0.37-1.1)	0.087
9d Thrombocytopenia	477	26 (22)	28 (23)	19 (16)	23 (19)	0.81 (0.42-1.5)	0.52
10 Immunological disorder	478	101 (85)	89 (74)	85 (71)	72 (61)	0.35 (0.18-0.66)	**0.0013**
10b Anti-DNA	478	97 (82)	85 (71)	75 (63)	61 (51)	0.28 (0.16-0.52)	**<0.0001**
10c Anti-Sm	478	41 (34)	21 (18)	37 (31)	25 (21)	0.73 (0.40-1.4)	0.32
11 ANA	478	117 (98)	118 (98)	119 (99)	118 (99)	2.7 (0.23-31)	0.43
**Autoantibody positivity**							
dsDNA	478	80 (67)	35 (29)	39 (33)	27 (23)	0.18 (0.10-0.32)	**<0.0001**
Nucleosome	478	83 (70)	44 (37)	53 (44)	35 (29)	0.23 (0.13-0.41)	**<0.0001**
Ribosomal P	478	14 (12)	10 (8.3)	6 (5.0)	2 (1.7)	0.14 (0.03-0.66)	**0.013**
Sm	478	32 (27)	18 (15)	27 (23)	13 (11)	0.52 (0.25-1.1)	0.082
Sm RNP	476	39 (33)	22 (18)	39 (33)	28 (24)	0.82 (0.45-1.5)	0.51
RNP A	476	42 (36)	23 (19)	34 (29)	25 (21)	0.57 (0.32-1.0)	0.069
RNP 68	478	15 (13)	7 (5.8)	20 (17)	9 (7.6)	0.84 (0.34-2.1)	0.71
SSA Ro52	478	36 (30)	26 (22)	30 (25)	39 (33)	1.0 (0.58-1.8)	0.96
SSA Ro60	477	57 (48)	45 (38)	39 (33)	55 (46)	0.80 (0.47-1.4)	0.41
SSB	478	23 (19)	23 (19)	28 (23)	31 (26)	1.4 (0.76-2.7)	0.27
β2GP1 IgG	477	32 (27)	38 (32)	27 (23)	23 (19)	0.62 (0.33-1.2)	0.14
β2GP1 IgM	478	9 (7.6)	13 (11)	2 (1.7)	8 (6.7)	0.81 (0.29-2.2)	0.68
β2GP1 IgA	477	22 (19)	27 (23)	14 (12)	14 (12)	0.54 (0.26-1.1)	0.10
ACL IgG	477	29 (24)	38 (32)	21 (18)	23 (19)	0.73 (0.39-1.4)	0.34
ACL IgM	477	9 (7.6)	8 (6.7)	2 (1.7)	5 (4.2)	0.48 (0.15-1.5)	0.21
ACL IgA	477	19 (16)	26 (22)	16 (13)	17 (14)	0.82 (0.40-1.7)	0.59

ACL: Anti-cardiolipin, ACR: American College of Rheumatology, ANA: Antinuclear antibody, dsDNA: double-stranded DNA, RNP: Ribonucleoprotein, Sm: Smith antigen, SSA/B: Sjögren's syndrome antigen A/B.

Supplementary Table 8. Associations between C4 and clinical manifestations.
C4 concentration was categorized into quartiles, and logistic regression models were used to calculate odds ratios (ORs) for disease manifestations and autoantibodies in the highest quartile (Q4) compared to the lowest quartile (Q1). Analyses were adjusted for sex and age at follow-up. P values <0.05, unadjusted for multiple comparisons, are shown in bold red.

	Total N	Q1 n (%)	Q2 n (%)	Q3 n (%)	Q4 n (%)	OR (95% CI)	p-value
**ACR 1982 classification criteria (ever)**							
1 Malar rash	505	61 (48)	61 (48)	63 (50)	59 (46)	1.0 (0.63-1.7)	0.90
2 Discoid rash	505	21 (17)	20 (16)	22 (18)	22 (17)	1.0 (0.52-2.0)	0.97
3 Photosensitivity	505	74 (58)	83 (66)	82 (66)	77 (61)	1.2 (0.72-2.0)	0.47
4 Oral ulcer	505	37 (29)	41 (33)	48 (38)	44 (35)	1.4 (0.84-2.5)	0.19
5 Arthritis	505	105 (83)	106 (84)	101 (81)	98 (77)	0.78 (0.42-1.5)	0.44
6 Serositis	505	47 (37)	44 (35)	50 (40)	58 (46)	1.3 (0.78-2.2)	0.32
6a Pleuritis	505	44 (35)	39 (31)	46 (37)	52 (41)	1.2 (0.70-2.0)	0.53
6b Pericarditis	504	21 (17)	20 (16)	19 (15)	28 (22)	1.3 (0.68-2.4)	0.43
7 Nephritis	505	65 (51)	45 (36)	41 (33)	51 (40)	0.60 (0.36-1.0)	0.050
8 Neurological disorder	505	10 (7.9)	14 (11)	18 (14)	13 (10)	1.3 (0.55-3.1)	0.55
8a Seizures	505	9 (7.1)	14 (11)	16 (13)	11 (8.7)	1.2 (0.49-3.1)	0.64
8b Psychosis	505	1 (0.8)	1 (0.8)	4 (3.2)	3 (2.4)	2.7 (0.28-27)	0.39
9 Haematological disorder	505	107 (84)	91 (72)	88 (70)	80 (63)	0.36 (0.19-0.65)	**0.00082**
9a Haemolytic anaemia	503	12 (9.4)	9 (7.1)	3 (2.4)	4 (3.1)	0.36 (0.11-1.2)	0.085
9b Leucopenia	505	76 (60)	63 (50)	64 (51)	41 (32)	0.33 (0.20-0.55)	**<0.0001**
9c Lymphopenia	505	88 (69)	66 (52)	62 (50)	51 (40)	0.33 (0.19-0.55)	**<0.0001**
9d Thrombocytopenia	505	35 (28)	28 (22)	16 (13)	19 (15)	0.42 (0.22-0.80)	**0.0080**
10 Immunological disorder	506	112 (88)	94 (75)	86 (68)	75 (59)	0.21 (0.11-0.40)	**<0.0001**
10b Anti-DNA	505	106 (84)	86 (69)	75 (59)	69 (54)	0.24 (0.13-0.43)	**<0.0001**
10c Anti-Sm	501	51 (40)	30 (24)	26 (21)	25 (20)	0.41 (0.23-0.73)	**0.0026**
11 ANA	506	126 (99)	124 (98)	125 (99)	125 (98)	0.57 (0.05-6.5)	0.65
**Autoantibody positivity**							
dsDNA	508	86 (68)	50 (39)	32 (25)	21 (17)	0.10 (0.05-0.18)	**<0.0001**
Nucleosome	508	93 (73)	59 (46)	36 (28)	36 (28)	0.15 (0.09-0.27)	**<0.0001**
Ribosomal P	508	17 (13)	16 (13)	2 (1.6)	2 (1.6)	0.11 (0.02-0.48)	**0.0036**
Sm	508	40 (31)	27 (21)	17 (13)	11 (9)	0.23 (0.11-0.48)	**0.00011**
Sm RNP	506	41 (33)	30 (24)	27 (21)	32 (25)	0.76 (0.44-1.3)	0.35
RNP A	489	42 (34)	33 (27)	24 (20)	23 (19)	0.49 (0.27-0.90)	**0.020**
RNP 68	508	14 (11)	15 (12)	11 (8.7)	10 (7.9)	0.81 (0.34-1.9)	0.63
SSA Ro52	508	43 (34)	30 (24)	37 (29)	29 (23)	0.54 (0.31-0.95)	**0.032**
SSA Ro60	507	67 (53)	44 (35)	55 (43)	44 (35)	0.44 (0.26-0.74)	**0.0019**
SSB	508	30 (24)	28 (22)	33 (26)	22 (17)	0.68 (0.36-1.3)	0.22
β2GP1 IgG	506	45 (35)	31 (24)	36 (29)	15 (12)	0.24 (0.13-0.47)	**<0.0001**
β2GP1 IgM	508	14 (11)	9 (7.1)	5 (3.9)	4 (3.1)	0.25 (0.08-0.78)	**0.017**
β2GP1 IgA	507	34 (27)	26 (20)	14 (11)	7 (5.5)	0.14 (0.06-0.34)	**<0.0001**
ACL IgG	506	40 (31)	31 (24)	31 (25)	14 (11)	0.27 (0.14-0.53)	**0.00016**
ACL IgM	507	13 (10)	6 (4.7)	4 (3.2)	2 (1.6)	0.13 (0.03-0.58)	**0.0079**
ACL IgA	507	31 (25)	26 (20)	15 (12)	10 (7.9)	0.25 (0.11-0.54)	**0.00039**

ACL: Anti-cardiolipin, ACR: American College of Rheumatology, ANA: Antinuclear antibody, dsDNA: double-stranded DNA, RNP: Ribonucleoprotein, Sm: Smith antigen, SSA/B: Sjögren’s syndrome antigen A/B.

Supplementary Table 9. Associations between C3 and clinical manifestations.
C3 concentration was categorized into quartiles, and logistic regression models were used to calculate odds ratios (ORs) for disease manifestations and autoantibodies in the highest quartile (Q4) compared to the lowest quartile (Q1). Analyses were adjusted for sex and age at follow-up. P values <0.05, unadjusted for multiple comparisons, are shown in bold red.

	Total N	Q1 n (%)	Q2 n (%)	Q3 n (%)	Q4 n (%)	OR (95% CI)	p-value
**ACR 1982 classification criteria (ever)**							
1 Malar rash	505	70 (55)	59 (46)	56 (45)	59 (47)	0.78 (0.47-1.3)	0.32
2 Discoid rash	505	20 (16)	26 (20)	21 (17)	18 (14)	0.77 (0.38-1.6)	0.47
3 Photosensitivity	505	79 (62)	72 (57)	83 (66)	82 (65)	1.2 (0.68-2.0)	0.60
4 Oral ulcer	505	39 (31)	48 (38)	43 (34)	40 (32)	1.2 (0.69-2.0)	0.55
5 Arthritis	505	111 (87)	103 (81)	98 (78)	98 (78)	0.55 (0.28-1.1)	0.085
6 Serositis	505	50 (39)	44 (35)	47 (38)	58 (46)	1.2 (0.71-2.0)	0.52
6a Pleuritis	505	45 (35)	39 (31)	43 (34)	54 (43)	1.2 (0.73-2.1)	0.45
6b Pericarditis	504	20 (16)	21 (17)	19 (15)	28 (22)	1.4 (0.74-2.7)	0.30
7 Nephritis	505	66 (52)	54 (43)	42 (34)	40 (32)	0.42 (0.25-0.71)	**0.0013**
8 Neurological disorder	505	12 (9.4)	16 (13)	12 (10)	15 (12)	1.2 (0.54-2.8)	0.62
8a Seizures	505	12 (9.4)	13 (10)	12 (10)	13 (10)	1.1 (0.47-2.5)	0.84
8b Psychosis	505	1 (0.8)	3 (2.4)	2 (1.6)	3 (2.4)	2.3 (0.23-23)	0.47
9 Haematological disorder	505	101 (80)	99 (78)	85 (68)	81 (64)	0.53 (0.30-0.94)	**0.029**
9a Haemolytic anaemia	503	8 (6.3)	11 (8.7)	6 (4.8)	3 (2.4)	0.43 (0.11-1.7)	0.23
9b Leucopenia	505	77 (61)	63 (50)	61 (49)	43 (34)	0.35 (0.21-0.58)	**<0.0001**
9c Lymphopenia	505	83 (65)	75 (59)	52 (42)	57 (45)	0.48 (0.29-0.80)	**0.0054**
9d Thrombocytopenia	505	34 (27)	26 (20)	20 (16)	18 (14)	0.41 (0.22-0.79)	**0.0076**
10 Immunological disorder	506	112 (89)	98 (77)	80 (63)	77 (61)	0.23 (0.11-0.44)	**<0.0001**
10b Anti-DNA	505	107 (85)	91 (72)	71 (57)	67 (53)	0.22 (0.12-0.41)	**<0.0001**
10c Anti-Sm	501	49 (40)	35 (28)	26 (21)	22 (17)	0.39 (0.21-0.71)	**0.0022**
11 ANA	506	125 (98)	127 (100)	123 (98)	125 (98)	1.3 (0.17-9.5)	0.82
**Autoantibody positivity**							
dsDNA	508	85 (67)	48 (38)	32 (25)	24 (19)	0.13 (0.07-0.23)	**<0.0001**
Nucleosome	508	93 (73)	54 (43)	39 (31)	38 (30)	0.18 (0.10-0.31)	**<0.0001**
Ribosomal P	508	17 (13)	11 (8.7)	6 (4.7)	3 (2.4)	0.17 (0.05-0.61)	**0.0064**
Sm	508	41 (32)	28 (22)	15 (12)	11 (8.7)	0.24 (0.12-0.51)	**0.00020**
Sm RNP	506	45 (36)	32 (25)	24 (19)	29 (23)	0.61 (0.35-1.1)	0.085
RNP A	489	45 (36)	31 (26)	26 (21)	20 (17)	0.39 (0.21-0.72)	**0.0024**
RNP 68	508	14 (11)	16 (13)	12 (9.4)	8 (6.3)	0.68 (0.27-1.7)	0.42
SSA Ro52	508	44 (35)	27 (21)	35 (28)	33 (26)	0.61 (0.35-1.1)	0.082
SSA Ro60	507	67 (53)	49 (39)	50 (39)	44 (35)	0.42 (0.25-0.71)	**0.0011**
SSB	508	30 (24)	24 (19)	29 (23)	30 (24)	1.0 (0.56-1.8)	1.0
β2GP1 IgG	506	38 (30)	41 (32)	31 (24)	17 (13)	0.35 (0.18-0.67)	**0.0014**
β2GP1 IgM	508	11 (8.7)	10 (7.9)	7 (5.5)	4 (3.1)	0.30 (0.09-0.99)	**0.048**
β2GP1 IgA	507	32 (25)	23 (18)	17 (13)	9 (7.1)	0.20 (0.09-0.45)	**<0.0001**
ACL IgG	506	35 (28)	37 (29)	29 (23)	15 (12)	0.35 (0.18-0.68)	**0.0021**
ACL IgM	507	9 (7.1)	9 (7.1)	5 (3.9)	2 (1.6)	0.18 (0.04-0.86)	**0.032**
ACL IgA	507	29 (23)	25 (20)	18 (14)	10 (7.9)	0.27 (0.12-0.58)	**0.00092**

ACL: Anti-cardiolipin, ACR: American College of Rheumatology, ANA: Antinuclear antibody, dsDNA: double-stranded DNA, RNP: Ribonucleoprotein, Sm: Smith antigen, SSA/B: Sjögren's syndrome antigen A/B.ACL: Anti-cardiolipin, ACR: American College of Rheumatology, ANA: Antinuclear antibody, dsDNA: double-stranded DNA, RNP: Ribonucleoprotein, Sm: Smith antigen, SSA/B: Sjögren's syndrome antigen A/B.

Supplementary Table 10. Associations with lead MAP-1 pQTL rs80288719 and SLE manifestations.

Patients were dichotomized as rs80288719 minor allele carriers (one or two copies of the minor allele) or non-carriers (two copies of the major allele). Logistic regression models were used to assess clinical phenotypes in carriers of the minor allele. Analyses were adjusted for sex and age at follow-up. P values <0.05, unadjusted for multiple comparisons, are shown in bold red.

	Total N	All n (%) n = 440	Carrier n (%) n = 61	Non-carrier n (%) n = 379	Chi² p-value	OR (95% CI)	p-value
**ACR 1982 classification criteria (ever)**							
1 Malar rash	437	220 (50)	28 (46)	192 (51)	0.45	0.84 (0.48-1.4)	0.52
2 Discoid rash	437	72 (17)	9 (15)	63 (17)	0.70	0.75 (0.35-1.6)	0.46
3 Photosensitivity	437	291 (67)	36 (59)	255 (68)	0.18	0.62 (0.35-1.1)	0.11
4 Oral ulcer	437	139 (32)	21 (34)	118 (31)	0.64	1.2 (0.68-2.2)	0.53
5 Arthritis	437	355 (81)	52 (85)	303 (81)	0.39	1.5 (0.71-3.3)	0.28
6 Serositis	437	172 (39)	28 (46)	144 (38)	0.26	1.3 (0.74-2.2)	0.38
6a Pleuritis	437	156 (36)	26 (43)	130 (35)	0.22	1.3 (0.75-2.3)	0.35
6b Pericarditis	436	75 (17)	15 (25)	60 (16)	0.085	1.7 (0.89-3.3)	0.11
7 Nephritis	437	172 (39)	23 (38)	149 (40)	0.78	1.0 (0.57-1.8)	0.99
8 Neurological disorder	437	49 (11)	10 (16)	39 (10)	0.17	1.6 (0.77-3.5)	0.20
8a Seizures	437	45 (10)	7 (12)	38 (10)	0.74	1.1 (0.48-2.7)	0.77
8b Psychosis	437	7 (1.6)	3 (4.9)	4 (1.1)	**0.026**	4.2 (0.90-20)	0.068
9 Haematological disorder	437	319 (73)	42 (69)	277 (74)	0.43	0.86 (0.47-1.6)	0.62
9a Haemolytic anaemia	436	25 (5.7)	1 (1.6)	24 (6.4)	0.14	0.26 (0.034-1.9)	0.19
9b Leucopenia	437	209 (48)	21 (34)	188 (50)	**0.024**	0.54 (0.31-0.95)	**0.033**
9c Lymphopenia	437	232 (53)	33 (54)	199 (53)	0.87	1.1 (0.63-1.9)	0.75
9d Thrombocytopenia	437	92 (21)	14 (23)	78 (21)	0.70	1.2 (0.60-2.2)	0.67
10 Immunological disorder	438	312 (71)	42 (69)	270 (72)	0.66	1.0 (0.56-1.9)	0.95
10b Anti-DNA	437	285 (65)	37 (62)	248 (66)	0.53	0.94 (0.53-1.7)	0.83
10c Anti-Sm	434	104 (24)	13 (21)	91 (24)	0.60	1.0 (0.51-2.0)	0.98
11 ANA	438	434 (99)	60 (98)	374 (99)	0.52	0.61 (0.060-6.2)	0.67
**Autoantibody positivity**							
dsDNA	440	159 (36)	19 (31)	140 (37)	0.38	0.90 (0.50-1.6)	0.73
Nucleosome	440	182 (41)	25 (41)	157 (41)	0.95	1.1 (0.65-2.0)	0.64
Ribosomal P	440	27 (6.1)	3 (4.9)	24 (6.3)	0.67	0.83 (0.24-2.9)	0.76
Sm	440	70 (16)	5 (8.2)	65 (17)	0.076	0.52 (0.20-1.4)	0.19
Sm RNP	438	102 (23)	11 (18)	91 (24)	0.30	0.77 (0.38-1.5)	0.46
RNP A	424	95 (22)	15 (25)	80 (22)	0.55	1.3 (0.67-2.4)	0.45
RNP 68	440	39 (8.9)	3 (4.9)	36 (9.5)	0.24	0.60 (0.18-2.0)	0.41
SSA Ro52	440	123 (28)	16 (26)	107 (28)	0.75	0.88 (0.47-1.6)	0.68
SSA Ro60	439	175 (40)	20 (33)	155 (41)	0.22	0.66 (0.37-1.2)	0.16
SSB	440	103 (23)	10 (16)	93 (25)	0.16	0.60 (0.29-1.2)	0.17
β2GP1 IgG	439	110 (25)	9 (15)	101 (27)	**0.045**	0.48 (0.23-1.0)	0.053
β2GP1 IgM	440	28 (6.4)	6 (9.8)	22 (5.8)	0.23	1.7 (0.66-4.4)	0.27
β2GP1 IgA	440	73 (17)	5 (8.2)	68 (18)	0.058	0.40 (0.15-1.0)	0.059
ACL IgG	439	100 (23)	7 (12)	93 (25)	**0.023**	0.40 (0.18-0.92)	**0.031**
ACL IgM	439	21 (4.8)	3 (4.9)	18 (4.8)	0.96	1.0 (0.28-3.5)	0.99
ACL IgA	440	74 (17)	7 (12)	67 (18)	0.23	0.60 (0.26-1.4)	0.23

ACL: Anti-cardiolipin, ACR: American College of Rheumatology, ANA: Antinuclear antibody, dsDNA: double-stranded DNA, MAP: Mannose-binding lectin-associated protein, pQTL: protein quantitative trait locus, RNP: Ribonucleoprotein, Sm: Smith antigen, SSA/B: Sjögren’s syndrome antigen A/B.

Supplementary Table 11. Associations of first independent MASP-2 pQTL rs72550870 with SLE manifestations.

Patients were dichotomized as rs72550870 minor allele carriers (one or two copies of the minor allele) or non-carriers (two copies of the major allele). Logistic regression models were used to assess clinical phenotypes in carriers of the minor allele. Analyses were adjusted for sex and age at follow-up. P values <0.05, unadjusted for multiple comparisons, are shown in bold red.

	Total N	All n (%) n = 440	Carrier n (%) n = 39	Non-carrier n (%) n = 401	Chi^2 p-value	OR (95% CI)	p-value
**ACR 1982 classification criteria (ever)**							
1 Malar rash	437	220 (50)	23 (59)	197 (50)	0.26	1.4 (0.72-2.7)	0.32
2 Discoid rash	437	72 (17)	4 (10)	68 (17)	0.27	0.58 (0.20-1.7)	0.32
3 Photosensitivity	437	291 (67)	26 (67)	265 (67)	0.99	0.94 (0.46-19)	0.87
4 Oral ulcer	437	139 (32)	9 (23)	130 (33)	0.22	0.58 (0.27-1.3)	0.18
5 Arthritis	437	355 (81)	31 (80)	324 (81)	0.77	0.82 (0.36-1.9)	0.64
6 Serositis	437	172 (39)	13 (33)	159 (40)	0.42	0.81 (0.40-1.6)	0.55
6a Pleuritis	437	156 (36)	12 (31)	144 (36)	0.50	0.84 (0.41-1.7)	0.64
6b Pericarditis	436	75 (17)	6 (15)	69 (17)	0.75	0.93 (0.37-2.3)	0.88
7 Nephritis	437	172 (39)	8 (21)	164 (41)	**0.012**	0.36 (0.16-0.81)	**0.014**
8 Neurological disorder	437	49 (11)	2 (5.1)	47 (12)	0.21	0.41 (0.094-1.7)	0.23
8a Seizures	437	45 (10)	2 (5.1)	43 (11)	0.27	0.45 (0.10-1.9)	0.28
8b Psychosis	437	7 (1.6)	1 (2.6)	6 (1.5)	0.62	1.9 (0.22-17)	0.54
9 Haematological disorder	437	319 (73)	31 (80)	288 (72)	0.34	1.4 (0.62-3.2)	0.42
9a Haemolytic anaemia	436	25 (5.7)	5 (13)	20 (5.0)	**0.046**	2.6 (0.92-7.5)	0.071
9b Leucopenia	437	209 (48)	22 (56)	187 (47)	0.26	1.4 (0.73-2.8)	0.30
9c Lymphopenia	437	232 (53)	25 (64)	207 (52)	0.15	1.6 (0.78-3.1)	0.21
9d Thrombocytopenia	437	92 (21)	10 (26)	82 (21)	0.46	1.3 (0.63-2.9)	0.45
10 Immunological disorder	438	312 (71)	29 (74)	283 (71)	0.65	1.1 (0.51-2.4)	0.82
10b Anti-DNA	437	285 (65)	23 (59)	262 (66)	0.39	0.69 (0.35-1.4)	0.29
10c Anti-Sm	434	104 (24)	14 (37)	90 (23)	0.052	1.8 (0.85-3.7)	0.13
11 ANA	438	434 (99)	38 (97)	396 (99)	0.26	0.25 (0.025-2.5)	0.24
**Autoantibody specificities**							
dsDNA	440	159 (36)	13 (33)	146 (36)	0.70	0.77 (0.38-1.6)	0.48
Nucleosome	440	182 (41)	19 (49)	163 (41)	0.33	1.3 (0.64-2.5)	0.50
Ribosomal P	440	27 (6.1)	2 (5.1)	25 (6.2)	0.78	0.75 (0.17-3.3)	0.70
Sm	440	70 (16)	11 (28)	59 (15)	**0.028**	2.1 (0.93-4.5)	0.075
Sm RNP	438	102 (23)	12 (32)	90 (23)	0.21	1.5 (0.71-3.1)	0.29
RNP A	424	95 (22)	11 (31)	84 (22)	0.18	1.6 (0.76-3.5)	0.21
RNP 68	440	39 (8.9)	6 (15)	33 (8.2)	0.13	1.9 (0.71-4.9)	0.20
SSA Ro52	440	123 (28)	12 (31)	111 (28)	0.68	1.2 (0.58-2.4)	0.65
SSA Ro60	439	175 (40)	17 (44)	158 (40)	0.62	1.2 (0.62-2.3)	0.59
SSB	440	103 (23)	11 (28)	92 (23)	0.46	1.3 (0.63-2.7)	0.48
β2GP1 IgG	439	110 (25)	13 (34)	97 (24)	0.17	1.6 (0.80-3.3)	0.18
β2GP1 IgM	440	28 (6.4)	4 (10)	24 (6.0)	0.30	1.8 (0.59-5.5)	0.30
β2GP1 IgA	440	73 (17)	9 (23)	64 (16)	0.25	1.6 (0.72-3.5)	0.24
ACL IgG	439	100 (23)	11 (29)	89 (22)	0.34	1.4 (0.67-3.0)	0.37
ACL IgM	439	21 (4.8)	3 (7.7)	18 (4.5)	0.37	1.8 (0.50-6.4)	0.38
ACL IgA	440	74 (17)	10 (26)	64 (16)	0.12	1.8 (0.85-3.9)	0.13

ACL: Anti-cardiolipin, ACR: American College of Rheumatology, ANA: Antinuclear antibody, dsDNA: double-stranded DNA, MASP: Mannose-binding lectin-associated serine protease, pQTL: protein quantitative trait locus, RNP: Ribonucleoprotein, Sm: Smith antigen, SSA/B: Sjögren's syndrome antigen A/B.

Supplementary Table 12. Associations of second independent MASP-2 pQTL rs1033638 with SLE manifestations.

Patients were dichotomized as rs1033638 minor allele carriers (one or two copies of the minor allele) or non-carriers (two copies of the major allele). Logistic regression models were used to assess clinical phenotypes in carriers of the minor allele. Analyses were adjusted for sex and age at follow-up. P values <0.05, unadjusted for multiple comparisons, are shown in bold red.

	Total N	All n (%) n = 440	Carrier n (%) n = 136	Non-carrier n (%) n = 304	Chi^2 p-value	OR (95% CI)	p-value
**ACR 1982 classification criteria (ever)**							
1 Malar rash	437	220 (50)	73 (54)	147 (49)	0.35	1.2 (0.82-1.9)	0.30
2 Discoid rash	437	72 (17)	26 (19)	46 (15)	0.32	1.3 (0.78-2.3)	0.29
3 Photosensitivity	437	291 (67)	89 (65)	202 (67)	0.73	0.96 (0.62-1.5)	0.86
4 Oral ulcer	437	139 (32)	38 (28)	101 (34)	0.24	0.78 (0.50-1.2)	0.27
5 Arthritis	437	355 (81)	105 (77)	250 (83)	0.15	0.71 (0.43-1.2)	0.18
6 Serositis	437	172 (39)	58 (43)	114 (38)	0.34	1.2 (0.79-1.8)	0.41
6a Pleuritis	437	156 (36)	55 (40)	101 (34)	0.16	1.3 (0.87-2.0)	0.20
6b Pericarditis	436	75 (17)	24 (18)	51 (17)	0.83	1.0 (0.60-1.8)	0.92
7 Nephritis	437	172 (39)	58 (43)	114 (38)	0.34	1.2 (0.79-1.8)	0.38
8 Neurological disorder	437	49 (11)	19 (14)	30 (10)	0.22	1.5 (0.80-2.7)	0.22
8a Seizures	437	45 (10)	17 (13)	28 (9.3)	0.31	1.4 (0.74-2.7)	0.31
8b Psychosis	437	7 (1.6)	2 (1.5)	5 (1.7)	0.88	0.93 (0.18-4.9)	0.94
9 Haematological disorder	437	319 (73)	94 (69)	225 (75)	0.22	0.77 (0.49-1.2)	0.25
9a Haemolytic anaemia	436	25 (5.7)	10 (7.4)	15 (5.0)	0.33	1.5 (0.67-3.5)	0.31
9b Leucopenia	437	209 (48)	66 (49)	143 (48)	0.84	1.1 (0.70-1.6)	0.82
9c Lymphopenia	437	232 (53)	67 (49)	165 (55)	0.28	0.82 (0.54-1.2)	0.34
9d Thrombocytopenia	437	92 (21)	28 (21)	64 (21)	0.87	0.95 (0.58-1.6)	0.85
10 Immunological disorder	438	312 (71)	89 (65)	223 (74)	0.072	0.67 (0.43-1.1)	0.077
10b Anti-DNA	437	285 (65)	83 (61)	202 (67)	0.22	0.77 (0.50-1.2)	0.23
10c Anti-Sm	434	104 (24)	29 (21)	75 (25)	0.38	0.83 (0.50-1.4)	0.47
11 ANA	438	434 (99)	134 (99)	300 (99)	0.41	0.46 (0.063-3.3)	0.44
**Autoantibody specificities**							
dsDNA	440	159 (36)	41 (30)	118 (39)	0.080	0.68 (0.44-1.1)	0.093
Nucleosome	440	182 (41)	51 (38)	131 (43)	0.27	0.80 (0.53-1.2)	0.32
Ribosomal P	440	27 (6.1)	6 (4.4)	21 (6.9)	0.31	0.63 (0.25-1.6)	0.34
Sm	440	70 (16)	19 (14)	51 (17)	0.46	0.83 (0.46-1.5)	0.53
Sm RNP	438	102 (23)	28 (21)	74 (24)	0.40	0.83 (0.50-1.4)	0.45
RNP A	424	95 (22)	29 (22)	66 (23)	0.84	0.97 (0.59-1.6)	0.90
RNP 68	440	39 (8.9)	13 (9.6)	26 (8.6)	0.73	1.2 (0.57-2.4)	0.67
SSA Ro52	440	123 (28)	39 (29)	84 (28)	0.82	1.1 (0.67-1.6)	0.83
SSA Ro60	439	175 (40)	48 (36)	127 (42)	0.22	0.77 (0.50-1.2)	0.22
SSB	440	103 (23)	37 (27)	66 (22)	0.21	1.4 (0.85-2.2)	0.20
β2GP1 IgG	439	110 (25)	27 (20)	83 (27)	0.10	0.67 (0.41-1.1)	0.11
β2GP1 IgM	440	28 (6.4)	7 (5.1)	21 (6.9)	0.48	0.74 (0.31-1.8)	0.50
β2GP1 IgA	440	73 (17)	21 (15)	52 (17)	0.67	0.88 (0.51-1.5)	0.66
ACL IgG	439	100 (23)	22 (16)	78 (26)	**0.031**	0.57 (0.34-0.96)	**0.034**
ACL IgM	439	21 (4.8)	6 (4.4)	15 (5.0)	0.81	0.89 (0.34-2.3)	0.82
ACL IgA	440	74 (17)	18 (13)	56 (18)	0.18	0.67 (0.38-1.2)	0.18

ACL: Anti-cardiolipin, ACR: American College of Rheumatology, ANA: Antinuclear antibody, dsDNA: double-stranded DNA, MASP: Mannose-binding lectin-associated serine protease, pQTL: protein quantitative trait locus, RNP: Ribonucleoprotein, Sm: Smith antigen, SSA/B: Sjögren's syndrome antigen A/B.

Supplementary Table 13. Associations of MASP-2 haploinsufficiency with SLE manifestations. Patients were dichotomized as rs72550870 or rs41307788 minor allele carriers (one or two copies of the minor alleles) or non-carriers (two copies of the major allele). Logistic regression models were used to assess clinical phenotypes in carriers of the minor allele. Analyses were adjusted for sex and age at follow-up. P values <0.05, unadjusted for multiple comparisons, are shown in bold red.

	Total N	All n (%) n = 440	Carrier n (%) n = 45	Non-carrier n (%) n = 395	Chi² p-value	OR (95% CI)	p-value
**ACR 1982 classification criteria (ever)**							
1 Malar rash	437	220 (50)	26 (58)	194 (50)	0.29	1.4 (0.73-2.6)	0.32
2 Discoid rash	437	72 (17)	4 (8.9)	68 (17)	0.15	0.49 (0.17-1.4)	0.19
3 Photosensitivity	437	291 (67)	30 (67)	261 (67)	0.99	1.0 (0.51-2.0)	0.99
4 Oral ulcer	437	139 (32)	10 (22)	129 (33)	0.15	0.56 (0.27-1.2)	0.13
5 Arthritis	437	355 (81)	36 (80)	319 (81)	0.82	0.88 (0.40-1.9)	0.76
6 Serositis	437	172 (39)	16 (36)	156 (39)	0.58	0.86 (0.45-1.7)	0.66
6a Pleuritis	437	156 (36)	15 (33)	141 (36)	0.73	0.93 (0.48-1.8)	0.82
6b Pericarditis	436	75 (17)	9 (20)	66 (17)	0.60	1.3 (0.58-2.8)	0.55
7 Nephritis	437	172 (39)	11 (24)	161 (41)	**0.031**	0.45 (0.22-0.92)	**0.028**
8 Neurological disorder	437	49 (11)	2 (4.4)	47 (12)	0.13	0.35 (0.081-1.5)	0.15
8a Seizures	437	45 (10)	2 (4.4)	43 (11)	0.17	0.38 (0.088-1.6)	0.19
8b Psychosis	437	7 (1.6)	1 (2.2)	6 (1.5)	0.73	1.8 (0.20-16)	0.61
9 Haematological disorder	437	319 (73)	34 (76)	285 (73)	0.68	1.1 (0.54-2.3)	0.76
9a Haemolytic anaemia	436	25 (5.7)	5 (11)	20 (5.1)	0.10	2.2 (0.79-6.3)	0.13
9b Leucopenia	437	209 (48)	24 (53)	185 (47)	0.44	1.3 (0.68-2.3)	0.47
9c Lymphopenia	437	232 (53)	27 (60)	205 (52)	0.33	1.3 (0.71-2.5)	0.37
9d Thrombocytopenia	437	92 (21)	13 (29)	79 (20)	0.17	1.6 (0.81-3.2)	0.18
10 Immunological disorder	438	312 (71)	34 (76)	278 (71)	0.50	1.2 (0.58-2.5)	0.61
10b Anti-DNA	437	285 (65)	28 (62)	257 (66)	0.66	0.82 (0.43-1.6)	0.55
10c Anti-Sm	434	104 (24)	14 (32)	90 (23)	0.20	1.4 (0.71-2.9)	0.32
11 ANA	438	434 (99)	44 (98)	390 (99)	0.33	0.31 (0.031-3.1)	0.32
**Autoantibody specificities**							
dsDNA	440	159 (36)	15 (33)	144 (37)	0.68	0.80 (0.41-1.6)	0.52
Nucleosome	440	182 (41)	21 (47)	161 (41)	0.45	1.2 (0.63-2.3)	0.58
Ribosomal P	440	27 (6.1)	3 (6.7)	24 (6.1)	0.88	1.0 (0.30-3.6)	0.94
Sm	440	70 (16)	11 (24)	59 (15)	0.098	1.7 (0.80-3.7)	0.17
Sm RNP	438	102 (23)	12 (27)	90 (23)	0.51	1.2 (0.59-2.5)	0.60
RNP A	424	95 (22)	12 (29)	83 (22)	0.27	1.5 (0.72-3.0)	0.28
RNP68	440	39 (8.9)	6 (13)	33 (8.4)	0.27	1.6 (0.62-4.1)	0.33
SSA Ro52	440	123 (28)	13 (29)	110 (28)	0.88	1.1 (0.54-2.1)	0.86
SSA Ro60	439	175 (40)	20 (44)	155 (39)	0.51	1.3 (0.67-2.4)	0.47
SSB	440	103 (23)	11 (24)	92 (23)	0.86	1.1 (0.52-2.2)	0.87
β2GP1 IgG	439	110 (25)	16 (36)	94 (24)	0.068	1.8 (0.94-3.5)	0.074
β2GP1 IgM	440	28 (6.4)	4 (8.9)	24 (6.1)	0.46	1.5 (0.51-4.7)	0.45
β2GP1 IgA	440	73 (17)	11 (24)	62 (16)	0.14	1.8 (0.84-3.6)	0.13
ACL IgG	439	100 (23)	14 (32)	86 (22)	0.13	1.7 (0.84-3.3)	0.14
ACL IgM	439	21 (4.8)	3 (6.7)	18 (4.6)	0.53	1.5 (0.43-5.4)	0.52
ACL IgA	440	74 (17)	12 (27)	62 (16)	0.062	2.0 (0.96-4.0)	0.066

ACL: Anti-cardiolipin, ACR: American College of Rheumatology, ANA: Antinuclear antibody, dsDNA: double-stranded DNA, MASP: Mannose-binding lectin-associated serine protease, RNP: Ribonucleoprotein, Sm: Smith antigen, SSA/B: Sjögren's syndrome antigen A/B.

Supplementary Table 14. Spearman's rank correlation coefficients with p-values for ficolin-3 activity and MASP-2, MAP-1, MASP-3, C4, C3, and C1q serum concentrations in controls and patients with SLE. P-values <0.05 are shown in bold red.

		Controls		SLE patients	
		ρ	p-value	ρ	p-value
Ficolin-3	MASP-2	0.42	**<0.0001**	0.45	**<0.0001**
	MAP-1	0.06	0.28	0.35	**<0.0001**
	MASP-3	-0.05	0.34	0.00	0.99
	C4	0.36	**<0.0001**	-0.02	0.71
	C3	0.37	**<0.0001**	0.01	0.77
	C1q	0.10	0.070	0.13	**0.0036**
MASP-2	MAP-1	0.00	0.99	0.18	**0.00011**
	MASP-3	0.01	0.82	0.03	0.52
	C4	0.24	**<0.0001**	0.05	0.25
	C3	0.26	**<0.0001**	0.15	**0.00089**
	C1q	0.04	0.45	0.09	0.06
MAP-1	MASP-3	0.26	**<0.0001**	0.29	**<0.0001**
	C4	-0.04	0.46	-0.10	**0.034**
	C3	-0.02	0.77	-0.10	**0.045**
	C1q	0.05	0.40	0.03	0.60
MASP-3	C4	-0.07	0.22	-0.04	0.39
	C3	-0.01	0.90	0.03	0.51
	C1q	0.20	**0.00032**	0.01	0.85
C4	C3	0.56	**<0.0001**	0.71	**<0.0001**
	C1q	0.24	**<0.0001**	0.45	**<0.0001**
C3	C1q	0.22	**<0.0001**	0.45	**<0.0001**
